# Physical Activity as a Habit in Long-Term Care: A Multidisciplinary Guideline

**DOI:** 10.3390/healthcare14050631

**Published:** 2026-03-02

**Authors:** Alyt Oppewal, Shanty Sterke, Stijn Weterings, Marscha M. Engelen, Tanja Mol

**Affiliations:** 1 Department of General Practice, Intellectual Disability Medicine Research, Erasmus MC, University Medical Center Rotterdam, 3015 GD Rotterdam, The Netherlands; 2 Academic Collaborative Research Center Healthy Ageing and Intellectual Disabilities, 3015 GD Rotterdam, The Netherlands; 3 Research Centre Innovations in Care, Rotterdam University of Applied Sciences, 3015 EK Rotterdam, The Netherlands; c.s.sterke@hr.nl; 4 Department of Physiotherapy, Aafje Nursing Homes, 3062 ME Rotterdam, The Netherlands; 5 Abrona, Healthcare Provider for Adults with Intellectual Disabilities, 3712 XA Huis ter Heide, The Netherlands; stijn.weterings@abrona.nl; 6 SKILZ, Institute for Developing Guidelines in Long-Term Care, 3528 BD Utrecht, The Netherlands; m.engelen@skilz.nu (M.M.E.); t.mol@skilz.nu (T.M.)

**Keywords:** exercise, healthcare, older persons, intellectual disabilities, nursing homes, home care

## Abstract

**Background:** Physical activity can help prevent and manage diseases, mental health conditions, and improve social connectedness and quality of life. However, integrating physical activity into long-term care settings remains a structural challenge. This paper presents a comprehensive overview of the systematic development and synthesis of a national, multidisciplinary guideline for integrating physical activity into routine long-term care practice. **Methods**: A multidisciplinary working group (*n* = 13) developed the guideline. A national online questionnaire (April–May 2023), disseminated through 20 organisations, identified and prioritised key challenges for implementing physical activity in long-term care. Next, critical questions were formulated and answered by systematic literature reviews, complemented with input from a focus group and sounding board groups, including all stakeholders. Recommendations were drafted and finalised through structured consensus procedures, integrating scientific evidence, stakeholder perspectives, contextual considerations, and professional expertise. **Results**: The guideline addressed the following critical questions: Why is physical activity important? How can care organisations best organise physical activity-focused care? How can care and support professionals integrate physical activity into the daily lives of clients? How and which physical activities can best be applied to stimulate physical activity? How can technology help? How can clients and their informal caregivers be motivated to engage in physical activity? Concrete recommendations were provided and remaining knowledge gaps identified. **Conclusions**: This guideline represents an important step towards embedding physical activity as a fundamental component of long-term care. It offers practical and evidence-informed recommendations for incorporating physical activity in routine long-term care practices, specifically addressing the unique challenges encountered in long-term care settings. The knowledge gaps can guide research to further support practice to normalise physical activity in long-term care.

## 1. Introduction

The health benefits of physical activity and exercise have been undeniably demonstrated in the general population. Regular physical activity can help prevent and manage a wide range of non-communicable diseases, including metabolic-, cardiovascular- and neurodegenerative diseases, various types of cancer, as well as mental health conditions such as anxiety and depression [1,2]. Moreover, physical activity plays a vital role in enhancing physical fitness, supporting musculoskeletal health, promoting healthy sleep, managing pain, fostering social connectedness and improving quality of life [2,3,4,5,6,7]. Physical activity and exercise are shown to be just as effective, and sometimes even more so, than medication in preventing and treating a wide range of conditions and in reducing the risk of premature death [2,8,9].

Globally, there is a growing awareness that physical activity and exercise can be used as medicine [10,11]. Nevertheless, global estimates indicate that 31% of adults fail to meet the recommended levels of physical activity [1]. Physical inactivity is still a key risk factor for non-communicable diseases and the fourth leading risk factor for premature mortality [12,13]. Despite various efforts, progress in increasing physical activity levels across populations remains limited. As a result, healthcare systems are increasingly burdened with preventable diseases, a burden that is expected to grow unless significant changes are made, leading to substantial economic, environmental, and societal costs [1]. The WHO is urging countries around the world to implement policy actions to reduce levels of physical inactivity and contribute to meeting the global target of a 15% relative reduction in insufficient physical activity by 2030 [1].

While many countries have developed national strategies and guidelines to promote physical activity, guidelines tailored to specific populations, such as individuals with disabilities or chronic conditions, and for distinct healthcare settings remain scarce [14,15]. As a result, integrating physical activity into healthcare systems and daily practices remains a structural challenge [16,17]. This is particularly evident in long-term care settings, such as nursing homes and services for individuals with (intellectual) disabilities, where the need for physical activity is especially urgent due to the high prevalence of physical inactivity and associated health issues [18,19,20,21]. This urgency is further underscored by recent evidence demonstrating that physical exercise can meaningfully improve function among older adults living in residential long-term care settings [21]. For these populations, engaging in physical activity is often complicated by tremendous personal, environmental and policy barriers they encounter, such as physical and cognitive limitations, comorbidities, the need for support, limited time and knowledge of care professionals and support staff, and the lack of inclusive physical activity opportunities. Physical activity interventions, programs and guidelines developed for and with the general population, and based on evidence in the general population, do not consider these specific barriers encountered in long-term care settings and the values, needs and preferences of individuals in long-term care. Guidelines for the general population are therefore unsuitable for individuals in long-term care who require a tailored approach [15].

Increasing physical activity levels in individuals in long-term care settings requires not only adaption to individual characteristics (e.g., physical and cognitive abilities), but also consideration of factors at the intrapersonal (e.g., availability of support, knowledge of support staff) and organisational level (e.g., availability of space and materials, a safe and motivating environment, policy), following the socio-ecological model [22]. These levels are interdependent: policies shape organisational priorities and resources, which influence staff practices and training, while staff attitudes and competencies directly affect individual opportunities and motivation. Conversely, the needs and experiences of individuals can inform professional practices and organisational strategies, ultimately driving policy adjustments. Therefore, a guideline tailored to the long-term care population and setting, incorporating all levels of the socio-ecological model, is essential to effectively promote physical activity in this population and facilitate the integration of physical activity and exercise into daily care practices. Existing international guidelines provide important general recommendations for promoting physical activity, such as the WHO guidelines on physical activity and sedentary behaviour [23] and the American College of Sports Medicine’s Expert Consensus Statement Prescribing Exercise and Designing Physical Activity Programs for People with Disabilities [15]. However, these primarily focus on physical activity prescriptions or programmatic advice, and do not provide a comprehensive, setting-wide framework for embedding physical activity into daily care routines within long-term care [14]. A guideline addressing all socio-ecological levels and designed for routine use in long-term care practice is still lacking.

In response to this need, this paper presents a comprehensive overview of the systematic development and synthesis of a national, multidisciplinary guideline for integrating physical activity into routine long-term care practice. In doing so, we provide a high-level description of the development process and a concise synthesis of the guideline’s core components, without presenting the full underlying systematic reviews or the complete guideline in detail.

## 2. Methods

This paper outlines the development process of this guideline in the Netherlands, highlights key challenges encountered by healthcare and support professionals, and presents the guideline’s key recommendations and identified knowledge gaps.

### 2.1. Setting and Target Group

The guideline is a single unified framework, targeted to individuals receiving primary, secondary or tertiary long-term care, due to (a combination of) psychogeriatric conditions, intellectual disabilities, gerontopsychiatric disorders, physical disabilities, and/or somatic conditions. Long-term care in this guideline is defined as care and support that is structural and often permanent, typically provided daily. This care and support focuses more on promoting or maintaining quality of life, rather than on curing illness. Long-term care is intended for individuals who, due to functional limitations, are dependent on care or support from others. This care can be provided at home or in healthcare facilities.

This guideline is primarily intended for all healthcare professionals (e.g., physiotherapists, movement therapists, occupational therapists, behavioural scientists, psychologists, social workers, physicians, advanced nurse practitioners, nurses, and support staff) who provide care and support to individuals receiving long-term care and who are involved in promoting physical activity as part of that care. Additionally, the knowledge and recommendations from the guideline can inform clients themselves, as well as their informal caregivers, such as relatives and volunteers. Finally, the guideline aims to inform and support care providers and policy makers in organising their care practices and in developing policies aimed at promoting physical activity.

### 2.2. Guideline Development

A multidisciplinary working group (*n* = 13) was formed to develop the guideline, consisting of different (para)medics (a general practitioner, an advanced nurse practitioner, physiotherapists, movement therapists, a psychologist, and an occupational therapist), scientists, a lifestyle coach, a support professional, and two process supervisors. All working group members declared their functions, ancillary positions, funding sources, and potential conflicts of interest prior to participation. These declarations were reviewed by SKILZ to assess eligibility and are publicly available in the published guideline documents.

The guideline was developed according to the methodology of SKILZ, the Dutch institute for developing guidelines in long-term care, which is in accordance with established standards for high-quality guideline development. The process was informed by the AQUA Framework (2021) [24], the HARING tools (2013) [25], and the AGREE II instrument (2010) [26].

#### 2.2.1. Needs Assessment of Key Challenges

Initially, relevant existing national and international guidelines, policy documents, and research related to the topic of physical activity in long-term care were identified through desk research, accompanied by interviews with two experts. Subsequently, a needs assessment was conducted to identify key challenges experienced with implementing physical activity in long-term care settings. This assessment was conducted using an online questionnaire. The questionnaire presented a list of challenges identified through the desk research, asking respondents to indicate whether they experienced these challenges. Additionally, respondents were asked to report any additional challenges encountered and indicate the top three challenges they believed should be prioritised in the guideline. Although the questionnaire was not formally validated, its structure has been applied in the development of multiple other guidelines by SKILZ and has demonstrated its utility in providing the input required to identify and prioritise the challenges that should be addressed in the guideline. The questionnaire was disseminated through a range of organisations, including professional associations (*n* = 11; e.g., occupational therapists, social workers, general practitioners, psychologists, intellectual disability physicians, specialists pedagogues, dietitians, physiotherapists, geriatric care physicians, nurses, and movement specialists), client organisations (*n* = 4; organisation for people with Alzheimer, people with intellectual disabilities, informal caregivers, and patients in general), and knowledge or advocacy institutes (*n* = 5; care providers for people with disabilities, healthcare insurers, the Centre for Sport and Physical Activity, and the academic collaborative research centers ‘Stronger on Your Own Feet’ initiative, and ’Healthy Ageing and Intellectual Disabilities’). In addition, the questionnaire was shared via the LinkedIn account of SKILZ. No predefined number of individuals was invited to participate. The questionnaire was open for responses from 3 April 2023 to 15 May 2023.

Respondents indicated the challenges they encountered and their top three priorities, which were analysed using simple frequency counts to determine how often each challenge was selected. These frequencies were used as input for a structured consensus discussion within the multidisciplinary working group, resulting in the key challenges to be addressed in the guideline. During this discussion, members considered the frequency data alongside the perceived relevance, feasibility, and expected impact of each challenge in long-term care practice. No weighted scoring system or statistical prioritisation method was applied. Instead, prioritisation was based on deliberative consensus, in accordance with SKILZ guideline development methodology.

#### 2.2.2. Critical Questions

Based on the outcomes of the online questionnaire, the prioritisation of the key challenges, and the expertise of the working group, critical questions underlying the key challenges were formulated. These critical questions formed the base of the guideline on which practical recommendations were provided. Recommendations were formulated based on different information sources.

Systematic literature reviews were performed following a predefined protocol, based on the PICO framework. Literature searches were performed in the databases CINAHL, Cochrane, Embase, PsycInfo and PubMed on 19 December 2023, covering articles published from January 2014 onwards. The search strategy and detailed inclusion and exclusion criteria are provided in Supplementary File A. Literature screening and selection were conducted in two stages by the team responsible for the literature reviews at SKILZ: title/abstract screening and full-text evaluation. All screening and selection steps were performed by one reviewer. A second reviewer independently screened and selected a random sample of 10% of the records. Any discrepancies were discussed until a consensus was reached. Risk of bias of included studies was assessed using appropriate tools, such as the AMSTAR-2 checklist [27] and risk-of-bias tables. Due to substantial heterogeneity across studies in terms of populations, interventions, outcomes, and study designs, no meta-analysis was performed. Instead, evidence was synthesised in evidence tables, and where possible, the certainty of evidence was assessed using the GRADE methodology [28] (Supplementary File B).

Additionally, input and feedback were provided via a focus group with managers and directors, and sounding board groups with stakeholders, including clients, client representatives, relatives and professional associations.

#### 2.2.3. Recommendations

The multidisciplinary working group formulated recommendations based on the scientific evidence, the input from the focus and sounding board groups, relevant contextual factors, and their own experience and expertise. To triangulate these information sources, each working group member received the systematic review results, as well as the outputs from the focus groups and sounding board groups. Members completed a questionnaire to synthesise these inputs with their professional experience and expertise and to draft initial recommendations. A consensus on the final recommendations was then achieved through structured deliberation during the working group meetings. This process followed the SKILZ guideline development methodology and did not involve a formal consensus technique. All 13 members of the multidisciplinary working group participated in this structured deliberation, drawing on their diverse professional backgrounds to ensure that the recommendations reflected clinical relevance, patient preferences, feasibility, ethical considerations, safety, and cost-effectiveness.

#### 2.2.4. Feedback and Authorisation

The focus group and sounding board groups provided feedback during the development and on the first concept of the guideline. After this, professional associations provided feedback, after which they authorised the guideline.

For the readability of this paper, the full systematic reviews and reports of the focus group and sounding board groups are not presented. A description of the included studies in the systematic reviews is presented in the Supplementary File (Figure S1, Tables S1–S3). The full systematic reviews and focus- and sounding board group reports are published with the Dutch guideline and can be found there (in Dutch; https://www.richtlijnenlangdurigezorg.nl/richtlijnen/bewegen-als-gewoonte-in-de-langdurige-zorg/overzicht-van-aanbevelingen accessed on 26 February 2026).

## 3. Results

### 3.1. Needs Assessment of Key Challenges

A total of 206 respondents filled out the online questionnaire for identifying key challenges for implementing physical activity in long-term care, with 194 respondents completing the final question of the survey. Respondents worked in different care settings (44% nursing home care for older adults, 49% intellectual disability care, 7% other) and were from 17 different professional backgrounds (27% nurse, 13% physiotherapist, 11% geriatric care physician, 10% support professional, 10% movement therapist, 8% occupational therapist, 21% other). As participation was voluntary and the questionnaire was disseminated through professional networks and social media, selection bias may have occurred, with a higher likelihood of responses from professionals who are already interested in or engaged with physical activity and guideline development.

### 3.2. Key Challenges

Table 1 presents how often the presented challenges were experienced by the respondents. Additional challenges mentioned by the respondents were lack of finances, staff shortage, lack of a suitable and inviting space for activities, lack of motivation of care and support professionals and informal caregivers, not knowing how to tailor activities, being uncertain or hesitant to take action because care is primarily organised around providing care and support with physical activity being perceived as an extra, and the lack of a clear policy for physical activity.

These challenges were then prioritised and categorised through consensus discussion within the guideline development working group, resulting in the key challenges that were addressed in the guideline (Table 2). The challenges about the lack of finances, staff and time and difficulties with transportation were chosen to not be separately addressed in the guideline, but were incorporated in the key challenges about ‘physical activity not being integrated in the daily routine’, as well as ‘the lack of knowledge about the potential benefits that increased physical activity and fitness of clients can bring to care and support professionals and care organisations’.

### 3.3. Critical Questions

Based on the identification and priorisation of the key challenges, underlying critical questions that had to be answered in the guideline were formulated by the working group and categorised into guideline modules (Table 2). For the modules ‘Organisation of care’, ‘Physical activities’, ‘Technology’, ‘Motivating clients’ and ‘Encouraging informal caregivers’, a systematic literature review was performed. A description of the included studies (*n* = 25) in these reviews is presented in Supplementary File B (Tables S1–S3), including the PRISMA flow chart (Figure S1), the search strategies, inclusion/exclusion criteria and risk of bias scores. The module ‘The importance of physical activity’ was based on existing literature reviews, key articles in the field and physical activity guidelines stating the benefits of physical activity. The working group decided that no additional systematic review was needed because these reviews, key articles and guidelines provided strong and recent evidence. For the module ‘A habit in daily life’, the working group decided not to perform a systematic review because the critical question for this module could best be answered by the practical expertise and experiences from the working group and the focus and sounding board groups.

### 3.4. Recommendations

For each module, the working group formulated recommendations based on the systematic literature reviews, input from the focus group and the sounding board groups, relevant contextual factors and their own experience and expertise.

#### 3.4.1. Module: The Importance of Physical Activity

The recommendations are:

- Physical activity has many health benefits and virtually no negative side effects. The most important benefit is the potential improvement in health, which may also be cost-effective.
- Physical activity has positive effects on physical health. It also positively influences mental health, well-being and social interactions. This can ultimately improve overall quality of life.
- Encouraging clients to be physically active can also have positive effects on care and support professionals and care organisations. It can reduce the workload, increase job satisfaction, and lower absenteeism.

#### 3.4.2. Module: Organisation of Care

The recommendations are:

- Establish policies on promoting physical activity within the care organisation. Ensure this includes a clear vision on physical activity and translates this policy into practical implementations.
- Collaborate with clients in the preparation, execution, and evaluation of policies related to physical activity promotion. This ensures the policy aligns with the motivation, wishes, and needs of clients.
- Embed the physical activity policy and its promotion into the organisational structure. Involve a physical activity ambassador and care and support professionals from all levels and departments within the organisation.
- Provide training and education for staff, volunteers, clients, and their relatives about the importance, benefits, and possibilities of physical activity.
- Document agreements on promoting physical activity and integrating it into daily life, for example, in the client’s care and support plan.
- Create opportunities within the care organisation’s environment that encourage clients, relatives, volunteers, and staff to be physically active.
- Collaborate with existing services and facilities in the local community.

#### 3.4.3. Module: A Habit in Daily Life

The recommendations are:

- Create opportunities for clients to participate in daily activities and enable them to be as independent as possible. Do not automatically take over tasks from the client. Be aware that what a client can do independently may vary from day to day and moment to moment.
- Enable clients to do what they are capable of themselves and expand this where possible. In the long term, this may help reduce workload and physical strain for care and support professionals (and relatives).
- Engage in conversations with clients about how physical activity can be more integrated into their daily lives, ensuring that activities align with what they enjoy doing.
- Use existing practical examples for inspiration on how and when physical activity can be incorporated into daily routines.

#### 3.4.4. Module: Physical Activities

The recommendations are:

- Create a plan together with the client (and, if applicable, their relatives) on how the client can become more physically active. Align with activities and sports that bring the client joy. Also consider physical activity that can take place during daily activities, as well as specific exercises to improve fitness and strengthen muscles. Keep evaluating the plan, as needs and abilities may change over time. If you are unable to figure it out together, include a movement specialist to help develop a plan to get the client more active.
- Take into account specific considerations for different client populations, tailored approaches are important when applying physical activities. Clients with specific needs may include clients with acquired brain injury, clients with (advanced) dementia, clients with severe and multiple disabilities, or clients who are wheelchair- or bed-bound;
- When implementing physical activity interventions, take into account the physical and cognitive abilities of clients. You can tailor physical activities by providing simple instructions and predictable exercises, demonstrating and repeating exercises regularly, offering exercises with varying levels of difficulty, choosing safe and easy exercises and ensuring a safe environment. Consider consulting with other disciplines to develop a plan for increasing the client’s physical activity.

#### 3.4.5. Module: Technology

The recommendations are:

- Consider using digital technology with the goal to engage clients in physical activity who would otherwise not be active without digital tools, to offer variety alongside analog activities, and to encourage clients to move more independently.
- Support clients in using digital technology. For example, take the lead in starting a digital game or assist the client with using a device.
- Guide and encourage volunteers and relatives in using digital technology (such as digital movement games) with the client.
- When using digital technology, take privacy and safety into account. For instance, do not enter client data into a system without proper consideration.
  Discuss with the client whether they understand the privacy and security settings. If in doubt, consider contacting the organisation’s IT staff.

#### 3.4.6. Module: Motivating Clients

The recommendations are:

- Make physical activity moments enjoyable for the client by exploring together what is suitable and fun.
- Consider incorporating a social component to encourage physical activity.
- Set positively formulated goals together that align with the client’s wishes. Break these goals down into achievable steps and avoid placing too much emphasis on “more physical activity”, instead, use physical activity as a means to reach meaningful goals.
- Provide appropriate tasks and clear, concrete instructions to clients.
- Encourage clients, reward physical activity, and celebrate successes.
- Together with clients, reflect on and appreciate the benefits of being active.
- Boost (self-)confidence and/or reduce fears related to physical activity among clients.

#### 3.4.7. Module: Encouraging Informal Caregivers

The recommendations are:

- Increase knowledge among relatives and volunteers about the benefits and possibilities of engaging in physical activity with clients;
- Boost enthusiasm among relatives and volunteers to be active together with clients;
- Strengthen the (self-)confidence of relatives and volunteers to engage in physical activity with the client and reduce fears of injury or falling.

### 3.5. Knowledge Gaps

For part of the critical questions, there was little or no research of good quality available. For these themes, the working group has formulated knowledge gaps for which future research is relevant to improve the scientific base of this guideline.

There is a need for more research on what helps clients in long-term care to become and remain more physically active over time. Most interventions in the literature have focused on the effect of physical activity interventions on health outcomes; however, less research has focused on interventions to increase physical activity levels. Further, the effects of technological interventions, such as digital games that incorporate physical activity, are inconsistent and require further research into physical and mental health outcomes. It is also unclear to what extent the use of these technologies increases physical activity.

The effects of increased physical activity in daily life on the physical, mental and social well-being of clients in long-term care are also not sufficiently studied. Studies so far often focus on larger-scale physical activity interventions, and not on smaller changes in daily life.

Finally, it is argued that increased physical activity will have a long-term impact on reducing the required care and associated healthcare costs (cost-effectiveness); however, this has not been adequately studied in the long-term care setting. Also, the effects of increased physical activity of clients on the workload, job satisfaction and absenteeism of care professionals and caregivers are insufficiently studied.

## 4. Discussion

While existing guidelines on promoting physical activity often focus on the general population, they rarely address the unique challenges encountered in long-term care settings. This paper describes the development of the Dutch multidisciplinary guideline ‘Physical activity as a habit in long-term care’. This guideline aims to support the long-term care setting to effectively promote physical activity and facilitate the integration of physical activity into daily care practices. From a multidisciplinary perspective, it offers concrete recommendations and advice to healthcare and support professionals and healthcare organisations. Best practices and scientific evidence are compiled and evaluated, resulting in a well-balanced, practical, and clear set of recommendations. This guideline adds value by explicitly integrating the socio-ecological perspective, addressing barriers at individual, intrapersonal, and organisational levels. It provides practical, context-specific recommendations tailored to the needs of individuals in long-term care and the professionals supporting them, bridging the gap between generic advice and real-world applicability in complex long-term care settings.

With physical inactivity still being a major risk factor for non-communicable diseases and the fourth leading risk factor for premature mortality [12,13], healthcare systems face an increasing burden from preventable diseases. This burden is expected to grow with the ageing of the population. The importance of physical activity in addressing these challenges can no longer be overlooked [1]. To effectively embed physical activity into healthcare systems and everyday practices, it is essential to develop population- and context-specific guidelines [14,16,17]. Although this guideline was developed within the Dutch long-term care context, we believe its recommendations have broader international relevance. The international scientific literature, policy documents and guidelines guided the identification of key challenges, and the available international scientific evidence guided the recommendations, making the guideline internationally relevant. However, its applicability outside the Netherlands will depend on local regulatory structures, staffing models, funding mechanisms and cultural norms, and therefore should be adapted rather than adopted wholesale. While we acknowledge that specific recommendations may need to be tailored to fit the unique circumstances of other countries and healthcare systems, this guideline can serve as a valuable starting point for developing localised approaches to promoting physical activity in long-term care settings.

This guideline emphasises the importance of integrating physical activity into daily care practices as a habitual and essential component, rather than treating it as an optional extra. This shift in perspective helps counter the frequently mentioned barriers of limited time and resources. By reframing care from something professionals *do for* clients to something they *enable clients to do themselves*, we align with the principles of function-focused care and reablement. Function-focused care promotes the preservation and optimisation of clients’ functional abilities by actively involving them in everyday tasks such as dressing, eating, walking, and personal hygiene [29,30]. Similarly, reablement aims to restore and maintain independence through short, intensive interventions that empower individuals to regain daily living skills [31,32,33]. Both approaches not only enhance clients’ autonomy and quality of life but also have the potential to reduce caregiver burden and improve job satisfaction among care professionals.

Although many countries have developed national strategies and guidelines to promote physical activity, these often lack specificity for particular populations and healthcare settings, such as individuals with disabilities or chronic conditions. Moreover, the implementation and effectiveness of such guidelines are generally rated as low to moderate [14]. The guideline ‘Physical activity as a habit in long-term care’ guideline contributes specifically by providing a multidisciplinary and system-oriented framework that translates both scientific evidence and stakeholder experience into actionable, context-specific recommendations for long-term care. It bridges the gap between general physical activity guidance and the practical realities of long-term care.

For guidelines to have a meaningful impact, their implementation is essential, requiring dedicated investments. Effective implementation begins with the involvement of all relevant stakeholders in the development of the guideline, ensuring shared ownership and commitment to its use [34]. In the case of the guideline ‘Physical activity as a habit in long-term care’, stakeholders were actively involved throughout its development and formally endorsed the guideline through their professional associations. These associations now play a key role in promoting and implementing the guideline within their domains. To ensure successful implementation within long-term care organisations, an effective management approach and targeted educational support are essential [35]. In addition, the presence of local champions (change agents) has proven to enhance adherence to guidelines [36].

To translate the practical recommendations of this guideline into practice, different stakeholder groups have distinct but interrelated responsibilities. Effective implementation requires a coordinated approach across individual, intrapersonal and organisational levels. Care and support professionals play a key role in integrating physical activity into daily care routines. This involves enabling clients to participate in everyday tasks, applying principles of function-focused care and reablement, and using motivational techniques. Managers are responsible for creating the organisational conditions necessary for sustainable change. This includes ensuring sufficient resources, allocating time for training, embedding physical activity in organisational vision and policy, and appointing movement ambassadors or other local champions. Relatives and informal caregivers can support clients by encouraging physical activity during visits, promoting continuity with the actions of healthcare and support professionals. Their involvement enhances consistency across settings and strengthens client motivation. Professional associations can promote adoption of the guideline by disseminating it among their members, integrating recommendations into training and continuing education, and monitoring adherence through visitations and quality assessments. Policy makers have an important role in creating enabling conditions at the system level. This includes embedding physical activity promotion in long-term care policy frameworks, funding implementation efforts, stimulating collaboration between organisations, and supporting the adoption of technologies that facilitate daily activity. By incentivising organisations to prioritise movement as part of basic care, policymakers can drive structural change across the sector. Collectively, these actions promote consistent and sustainable implementation of the guideline and support the integration of physical activity as a standard component of daily long-term care. These implementation considerations align with recognised implementation frameworks, such as the Consolidated Framework for Implementation Research (CFIR), which emphasises multilevel determinants, including individuals, inner setting, outer setting, and processes, that directly map onto the socio-ecological levels addressed in this guideline.

Based on the systematic literature reviews that were performed, knowledge gaps were identified that require further research to strengthen the scientific base of the recommendations. To guide future work, a distinction can be made between short-term and long-term priorities. In the short term, research should focus on understanding which strategies effectively support clients to become and remain more physically active over time, and how technology can facilitate activity in long-term care. Further, the effects of increased physical activity in daily life on the physical, mental and social well-being of clients in long-term care should be studied. Long-term priorities include evaluating the long-term impact of increased physical activity of clients on reducing the required care and associated healthcare costs (cost-effectiveness), and workforce outcomes such as workload, job satisfaction and absenteeism. For policymakers, a suggested research agenda includes investing in implementation studies, stimulating cross-sector collaboration, supporting technological innovation, and funding long-term evaluation studies that can inform structural policy adjustments.

While this guideline fills an important gap in supporting long-term care settings to promote physical activity and integrate physical activity into daily care practices, this guideline also has some limitations. The guideline was developed within the Dutch long-term care context, which may limit its direct applicability in other countries with different organisational healthcare structures, resources, and cultural norms. Although international evidence informed the recommendations, some may require adaptation to local contexts before use (e.g., recommendations in the module ‘Organisation of care’ may need adaptation for different healthcare structures). Furthermore, the evidence base for certain recommendations is limited, and therefore some rely more on expert consensus, due to gaps in research on long-term effects and cost-effectiveness.

## 5. Conclusions

In conclusion, this guideline represents a unique contribution by providing a multidisciplinary and system-oriented framework specifically tailored to long-term care settings, supporting the embedding of physical activity as a fundamental component of long-term care. By addressing key challenges and involving all relevant stakeholders in its development, it offers practical and evidence-informed recommendations for incorporating physical activity in routine long-term care practices. The development process integrated multiple sources of evidence (systematic reviews, stakeholder input, and expert consensus) and used a structured consensus methodology, focusing on system-level integration. The resulting recommendations therefore reflect the best available evidence combined with professional judgement where evidence was limited.

While the guideline is rooted in the Dutch context, its multidisciplinary approach and alignment with international evidence make it a valuable reference for other countries seeking to enhance physical activity in long-term care settings. The guidelines principles, such as the socio-ecological approach and the focus on daily-life activity, are informative for other countries. However, international applicability requires careful contextual adaptation. To fully realise its potential, continued efforts are needed to support implementation, monitor outcomes, and address the identified knowledge gaps through targeted research. Only then can physical activity truly become a sustainable habit in long-term care, benefiting clients, professionals, and healthcare systems alike.

## Figures and Tables

**Table 1:** Percentage of participants in the online questionnaire that experienced the presented challenges.

Presented Challenges	% Experienced
Too little time available for care and support professionals.	84.0%
Difficulties with motivating the client.	75.7%
Lack of awareness among care and support professionals about the benefits of and possibilities for physical activity.	65.1%
Lack of knowledge among care and support professionals about suitable physical activity interventions.	62.1%
Lack of tailored activities.	57.3%
Difficulties with performing physical activities with clients and incorporating physical activity in daily practice.	44.0%
Lack of knowledge among care and support professionals about using technologies.	41.3%
Difficulties with transportation.	37.9%
The facility/environment does not encourage physical activity.	33.5%
Existing recommendations and exercises are not feasible for the target population.	28.2%

**Table 2:** Key challenges, underlying critical questions, the modules of the guideline ‘Physical activity as a habit in long-term care’, and the underlying information sources used to formulate the recommendations. For all modules, expertise from the working-, focus- and sounding board group were used as an information source.

Key Challenges	Underlying Critical Questions	Module (Information Sources)
Lack of knowledge and awareness about benefits of and opportunities for physical activity. Lack of knowledge about the potential benefits that increased physical activity and fitness of clients can bring to care and support professionals. Uncertainty or hesitation in taking action because care is primarily organised around providing care and support, with physical activity being often perceived as an “extra.”	Why is physical activity important for clients receiving long-term care?	The importance of physical activity (existing reviews, key articles and physical activity guidelines)
The facility/environment does not encourage physical activity. Lack of a suitable and inviting space for activities. Lack of knowledge about the potential benefits that increased physical activity and fitness of clients can bring to care organisations.	How can long-term care organisations best organise physical activity-focused care to encourage and normalise physical activity among clients?	Organisation of care (systematic review *n* = 11 and existing key articles)
Physical activity is not integrated into the daily routine.	How can care and support professionals integrate physical activity into the daily lives of clients receiving long-term care?	A habit in daily life (existing reviews, key articles and physical activity guidelines)
Too few tailored activities available, and not knowing how to tailor activities and physical activity recommendations to the abilities and wishes of the client.	How and which physical activities can best be applied to stimulate physical activity in clients receiving long-term care?	Physical activities (systematic review *n* = 6)
Lack of knowledge about how technology can help and what is available.	How can technology best be used to encourage physical activity in clients receiving long-term care?	Technology (systematic review *n* = 8)
Lack of motivation among care and support professionals. Lack of motivation in clients.	How can clients in long-term care be best motivated to be more physically active?	Motivating clients (systematic review *n* = 11)
Lack of motivation and stimulation of informal (e.g., relatives, volunteers) caregivers.	How can informal caregivers and the surrounding environment in long-term care be best motivated to engage in physical activity together with clients?	Encouraging informal caregivers (systematic review *n* = 11)

## Data Availability

The guideline, including the results of the systematic literature reviews and a detailed version of the results of the online questionnaire (in Dutch), can be found at www.richtlijnenlangdurigezorg.nl, accessed on 26 February 2026. More information is available from the corresponding author upon reasonable request.

## Supplementary Materials

The following supporting information can be downloaded at: https://www.mdpi.com/article/10.3390/healthcare14050631/s1: Supplementary File A: Methods. Supplementary File B: Results. Figure S1: Flow diagram of study selection in the systematic reviews. Tables S1–S3: Study characteristics of included studies in the systematic reviews.
