# Supplementary material for: Physical Activity as a Habit in Long-Term Care: A Multidisciplinary Guideline
Source: Healthcare (Basel). 2026 Mar 2;14(5):631. doi: 10.3390/healthcare14050631 (PMC12984252; doi:10.3390/healthcare14050631)
Supplement: Supplementary file 1 [file healthcare-14-00631-s001.zip › healthcare-4150618-SI.pdf]

Physical activity as a habit in long-term care: A multidisciplinary guideline

Supplementary files

Supplementary file A: Methods

Search strategy

Broad searches were conducted for the systematic reviews for all research questions related to the guideline in the databases CINAHL, Cochrane, Embase, PsycInfo and Pubmed. Box 1 provides the PubMed search string; equivalent search strings were used in the other databases.

Search date: 19 December 2023

#	Searches
#1	"Long-Term Care"[Mesh] OR "long-term car*"[tiab] OR "longterm car*"[tiab] OR "longer term car*"[tiab] OR "chronic care"[tiab] OR "continuous care"[tiab] OR "Residential Facilities"[Mesh:noexp] OR "residential facilit*"[tiab] OR "residential car*"[tiab] OR "care facilit*"[tiab] OR "residential aged care"[tiab] OR "assisted living facilities"[Mesh] OR "assisted living"[tiab] OR "group homes"[Mesh] OR "group home*"[tiab] OR "group living home*"[tiab] OR "Homes for the Aged"[Mesh] OR "home for the ag*"[tiab] OR "Nursing Homes"[Mesh] OR "Home nursing"[Mesh] OR "home health nursing"[Mesh] OR "nursing home*"[tiab] OR "home nurs*"[tiab] OR "nursing facilit*"[tiab] OR "home health nurs*"[tiab] OR "Home care services"[Mesh:noexp] OR "home car*"[tiab] OR "care service*"[tiab] OR "adult day care centers"[Mesh] OR "adult day care center*"[tiab] OR "day car*"[tiab] OR "Adolescent, Institutionalized"[Mesh] OR "adolescent institutionalized"[tiab] OR "institutionalized adolescent*"[tiab] OR "housing for the elderly"[Mesh] OR "housing for the elder*"[tiab] OR "life care center*"[tiab] OR "continuing care retirement center*"[tiab] OR "palliative care"[Mesh] OR "palliative car*"[tiab]
#2	“multiple chronic conditions"[Mesh] OR "multiple chronic condition*"[tiab] OR "multiple chronic illness*"[tiab] OR "multimorbidity"[Mesh] OR "multimorbidit*"[tiab] OR (("end of life"[tiab] OR "end stag*"[tiab] OR "stage 4"[tiab] OR "stage four"[tiab] OR "stage 5"[tiab] OR "stage five"[tiab] OR "advanced"[tiab] OR "secondary progressive*"[tiab] OR "fully dependen*"[tiab] OR "very dependen*"[tiab] OR "severe"[tiab]) AND ("Parkinson Disease"[Mesh] OR "Parkinson"[tiab] OR "parkinson disease, secondary"[Mesh] OR "heart failure"[Mesh] OR "heart failure"[tiab] OR "heart decompensation"[tiab] OR "myocardial failure*"[tiab] OR "osteoporosis"[Mesh] OR "osteoporosis"[tiab] OR "bone loss"[tiab] OR "pulmonary disease, chronic obstructive"[Mesh] OR "pulmonary disease, chronic obstructive"[tiab] OR "asthma-chronic"[tiab] OR "bronchitis chronic"[tiab] OR "pulmonary emphysema"[tiab] OR "chronic obstructive lung disease*"[tiab] OR "chronic airway obstruction*"[tiab] OR "stroke"[Mesh] OR "stroke*"[tiab] OR "cerebrovascular accident*"[tiab] OR "vascular accident brain*"[tiab] OR "brain infarction"[tiab] OR "hemorrhagic stroke"[tiab] OR "ischemic"[tiab] OR "poststroke"[tiab] OR "multiple system atrophy"[Mesh] OR "Multiple System Atrophy"[tiab] OR "amyotrophic lateral sclerosis"[Mesh] OR "amyotrophic lateral sclerosis "[tiab] OR "gehrig’s disease"[tiab] OR "multiple sclerosis"[Mesh] OR "multiple sclerosis"[tiab] OR "MS"[tiab] OR "disseminated sclerosis"[tiab] OR "multiple sclerosis chronic progressive"[tiab] OR "multiple sclerosis relapsing-remitting"[tiab] OR "diabetes mellitus"[Mesh] OR "diabetes mellitus"[tiab] OR "brain injury, chronic"[Mesh] OR "chronic brain injur*"[tiab] OR "chronic traumatic encephalopath*"[tiab]))
#3	"Intellectual Disability"[Mesh] OR "intellectual disab*"[tiab] OR "intellectual development disorder*"[tiab] OR "idiocy"[tiab] OR "intellectual retard*"[tiab] OR "intellectual defici*"[tiab] OR "intellectual

	handicap*"[tiab] OR "intellectual impair*"[tiab] OR "intellectual incapacit*"[tiab] OR "intellectually retarded"[tiab] OR "intellectually disabled"[tiab] OR "intellectually handicapped"[tiab] OR "intellectually impaired"[tiab] OR "intellectual disorder*"[tiab] OR "intellectual dysfunction*"[tiab] OR "Persons with Mental Disabilities"[Mesh] OR "mental retard*"[tiab] OR "mental defici*"[tiab] OR "mental disab*"[tiab] OR "mental handicap*"[tiab] OR "mental impair*"[tiab] OR "mental incapacit*"[tiab] OR "mentally retard*"[tiab] OR "mentally disabl*"[tiab] OR "mentally handicap*"[tiab] OR "mentally impaired"[tiab] OR "mentally incapacitated"[tiab] OR "cognitive retard*"[tiab] OR "Cri-du-Chat Syndrome*"[tiab] OR "De Lange Syndrome*"[tiab] OR "Down Syndrome*"[tiab] OR "mongolism"[tiab] OR "Down's syndrome*"[tiab] OR "Downs syndrome*"[tiab] OR "trisomy G"[tiab] OR "Adrenoleukodystrophy"[tiab] OR "Coffin-Lowry Syndrome*"[tiab] OR "Coffin Syndrome*"[tiab] OR "Fragile X Syndrome*"[tiab] OR "FRAXE syndrome*"[tiab] OR "marker X syndrome*"[tiab] OR "glycogen storage disease type IIb"[tiab] OR "lesch-nyhan syndrome*"[tiab] OR "menkes kinky hair syndrome*"[tiab] OR "Mucopolysaccharidosis II*"[tiab] OR "Mucopolysaccharidosis 2*"[tiab] OR "sanfilippo syndrome*"[tiab] OR "pyruvate dehydrogenase complex deficiency disease*"[tiab] OR "hunter syndrome*"[tiab] OR "Rett Syndrome*"[tiab] OR "rett disorder*"[tiab] OR "Prader-Willi"[tiab] OR "prader willi"[tiab] OR "labhart-willi syndrome*"[tiab] OR "labhart willi syndrome*"[tiab] OR "willi prader syndrome*"[tiab] OR "royer syndrome*"[tiab] OR "Rubinstein-Taybi Syndrome*"[tiab] OR "broad thumb-hallux syndrome*"[tiab] OR "rubinstein syndrome*"[tiab] OR "patau syndrome*"[tiab] OR "trisomy 13"[tiab] OR "patau's syndrome*"[tiab] OR "WAGR Syndrome*"[tiab] OR "WAGR complex"[tiab] OR "Williams Syndrome*"[tiab] OR "beuren syndrome*"[tiab] OR "Williams-beuren syndrome*"[tiab] OR "chromosome disorders"[Mesh] OR "chromosome disorder*"[tiab] OR "22q11 Deletion Syndrome*"[tiab] OR "Angelman Syndrome*"[tiab] OR "happy puppet"[tiab] OR "holoproscencephaly"[tiab] OR "11q deletion syndrome*"[tiab] OR "Smith-Magenis Syndrome*"[tiab] OR "Sotos Syndrome*"[tiab] OR "trisomy 18 syndrome*"[tiab] OR "wolf-hirschhorn syndrome*"[tiab] OR "Pitt-Hopkins syndrome*"[tiab] OR "CDKL5 deficiency disorder*"[tiab] OR "Tuberous Sclerosis"[Mesh] OR "Tuberous Sclerosis*"[tiab] OR "Noonan Syndrome"[Mesh] OR "Noonan Syndrome*"[tiab] OR "CHARGE Syndrome"[Mesh] OR "CHARGE Syndrome*"[tiab] OR "Smith-Lemli-Opitz Syndrome"[Mesh] OR "Smith-Lemli-Opitz Syndrome*"[tiab]
#4	"Geriatrics"[Mesh] OR "geriatric*"[tiab] OR "gerontology"[tiab] OR "geroscience"[tiab] OR "neurocognitive disorders"[Mesh] OR "neurocognitive disorder*"[tiab] OR "organic mental disorder*"[tiab] OR "Dementia"[MeSH] OR "Dementia*"[tiab] OR "Amentia*"[tiab] OR "Alzheimer*"[tiab] OR "creutzfeld-jakob*"[tiab] OR "CJD"[tiab] OR "subacute spongiform encephalopath*"[tiab] OR "binswanger*"[tiab] OR "huntington*"[tiab] OR "kluver-bucy syndrome*"[tiab] OR "lewy body disease*"[tiab] OR "korsakoff syndrome"[Mesh] OR "korsakoff syndrome"[tiab] OR "korsakoff psych*"[tiab]
#5	#1 or #2 or #3 or #4
#6	exercise[Mesh] OR exercise therapy[Mesh] OR sports for persons with disabilities[Mesh] OR exercis*[title] OR physical activity[title] OR physical exertion[title] OR movement[title] OR workout*[title] OR physical training[title] OR strength training[title] OR physical rehabilitation[title] OR sport*[title]
#7	("Meta-Analysis"[Publication Type] OR "Meta-Analysis as Topic"[Mesh] OR metaanaly*[tiab] OR meta-analy*[tiab] or metanaly*[tiab] OR "Systematic Review" [Publication Type] OR systematic[sb] OR "Cochrane Database Syst Rev"[Journal] or prisma[tiab] OR preferred reporting items[tiab] OR prospero[tiab] OR ((systemati*[ti] OR scoping[ti] OR umbrella[ti] OR structured literature[ti]) AND (review*[ti] OR overview*[ti])) OR systematic review*[tiab] OR scoping review*[tiab] OR umbrella review*[tiab] OR structured literature review*[tiab] OR systematic qualitative review*[tiab] OR systematic quantitative review*[tiab] OR systematic search and review[tiab] OR systematized review[tiab] OR systematised review[tiab] OR systemic review[tiab] OR systematic literature review*[tiab] OR systematic integrative literature review*[tiab] OR systematically review*[tiab] OR scoping literature review*[tiab] OR systematic critical review[tiab] OR systematic integrative review*[tiab] OR systematic evidence review[tiab] OR Systematic integrative literature review*[tiab] OR Systematic mixed studies review*[tiab] OR Systematized literature review*[tiab] OR Systematic overview*[tiab] OR Systematic narrative review*[tiab] OR ((systemati*[tiab] OR literature[tiab] OR database*[tiab] OR data-base*[tiab] OR structured[tiab] OR

	comprehensive*[tiab] OR systemic*[tiab]) AND search*[tiab]) OR (Literature[ti] AND review[ti] AND (database*[tiab] OR data-base*[tiab] OR search*[tiab])) OR ((data extraction[tiab] OR data source*[tiab]) AND study selection[tiab]) OR (search strategy[tiab] AND selection criteria[tiab]) OR (data source*[tiab] AND data synthesis[tiab]) OR medline[tiab] OR pubmed[tiab] OR embase[tiab] OR Cochrane[tiab] OR ((critical[ti] OR rapid[ti]) AND (review*[ti] OR overview*[ti] OR synthes*[ti])) OR (((critical*[tiab] OR rapid*[tiab]) AND (review*[tiab] OR overview*[tiab] OR synthes*[tiab]) AND (search*[tiab] OR database*[tiab] OR data-base*[tiab]))) OR metasynthes*[tiab] OR meta-synthes*[tiab]) NOT ("Comment" [Publication Type] OR "Letter" [Publication Type] OR "Editorial" [Publication Type] OR (("Animals"[Mesh] OR "Models, Animal"[Mesh]) NOT "Humans"[Mesh]))
#8	#5 and #6 and #7

Inclusion and exclusion criteria

The literature was systematically selected based on predefined criteria for all research questions. The inclusion criteria were as follows:

- **Year of publication:** Articles published from 1 January 2014 onwards.
- **Language:** Dutch and English.
- **Country:** Participants residing in a first- or second-world country.
- **Study designs:** Systematic literature reviews (meta-analyses, systematic reviews, scoping reviews, etc.).
- **Target population:** Clients aged 18 years and older receiving long-term care (at home or in an institutional setting).

Literature was excluded if:

- The study population consisted of healthy individuals, individuals not receiving long-term care, or individuals receiving care in hospital settings, medical specialist rehabilitation, or only a light form of informal care.
- In more than 50% of the included articles within a review, it was unclear whether long-term care was provided or how severe the condition was.
- Participants resided in a third-world country ("low-income countries").
- **Study designs:** Individual studies (randomized controlled trials, cohort studies, case–control studies, etc.), descriptive studies, conference abstracts, protocols, or case reports.
    - Studies with fewer than 10 participants.
- Children (<18 years) were included or age was not reported; or if more than 50% of the included articles within a review focused on children (18 years or younger).

Supplementary file B: Results

Figure S1: Flow diagram of study selection in the systematic reviews.

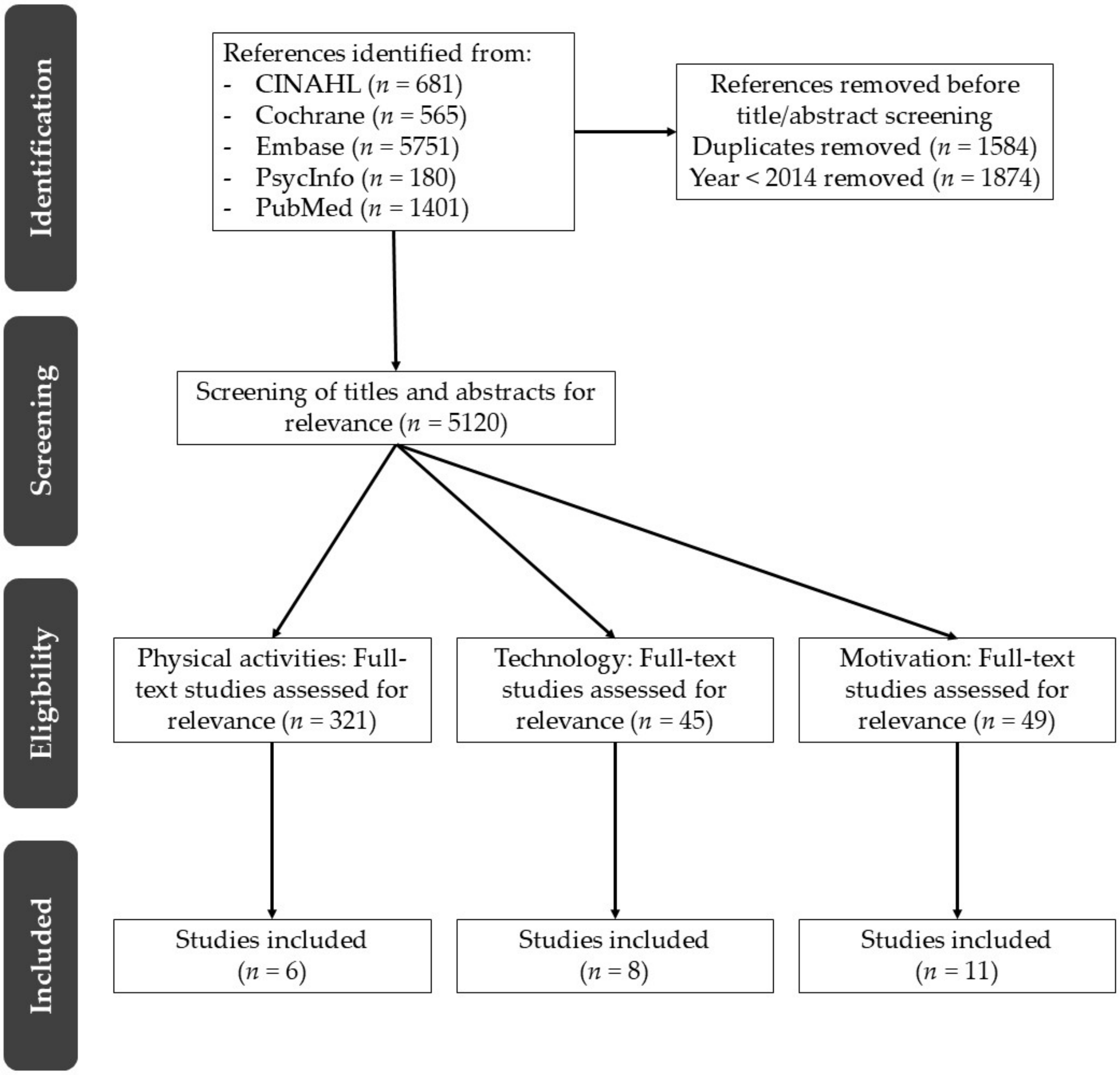

Tables S1 – S3: Study characteristics of included studies in the systematic reviews.

Table S1: Data extraction of included studies on physical activities (n=6; module 'Physical activities').

Author, publication year: Barrett et al. 2021'
Review aim: Effectiveness of Functionally based Physical Activity Programs on Physical, Psychological, Cognitive, and Adverse Outcomes in Older Adults Living in Nursing Homes: Systematic Review

**Review aim**	**Review characteristics** *(databases, search date)*	**Characteristics of included studies** (number of studies, design, country, setting)	**Patient characteristics**	**Interventions and comparisons**	**Outcome measures and effect size**	**Conclusion**	**Comments**
Effect of function-oriented PA programs on patients with **AD** or **dementia in nursing homes.**	CINAHL, Cochrane Database, EMBASE, MEDLINE, PsychInfo, PubMed, and Web of Science, June 2018.	N=23, RCTs, mostly western countries, nursing homes.	Older adults, mean age NR with **AD or dementia** diagnosis.	Intervention:<br><br>- Walking through the corridors.<br>- Walking sessions between 15 to 30 mins.<br><br>Controls:<br>- Usual care.<br>- No intervention.	PA:<br>- Adherence to walking: + improved at 16 weeks.<br>- Daily PA: - no effect.	The review reports some evidence to support positive effect of walking programs on PA.	+ PA

Author, publication year: Brett et al. 2016
Review aim: Effects of Physical Exercise on Health and Well-Being of Individuals Living With a Dementia in Nursing Homes: A Systematic Review

**Review aim**	**Review characteristics** *(databases, search date)*	**Characteristics of included studies** (number of studies, design, country, setting)	**Patient characteristics**	**Interventions and comparisons**	**Outcome measures and effect size**	**Conclusion**	**Comments**
Effect of physical exercise on persons with	Academic Search Complete, British Medical Journal Database, Cochrane	N=17, RCTs, Europe, North and South America,	N=901, mean age 82.6, persons with **dementia.**	Intervention:<br>- Multimodel exercise.<br>- Walking.	PA:<br>- Activity level. No significant findings.	Emerging evidence on effect of physical exercise on health.	- PA

**dementia in nursing homes.**	Library, Informa Nursing Consult, Informit, PEDro, Proquest Central, PubMed, Science Direct, Scopus, and Web of Science, search date NR.	Asia, nursing homes.		- Music and movement.<br>- Hand exercise.<br>Control:<br>- Interaction through social visits and conversations.<br>- Reading.			
**Author, publication year: Brooker et al. 2015**<br>**Review aim: Systematic Review of Interventions Aiming to Improve Involvement in Physical Activity Among Adults With Intellectual Disability**							
**Review aim**	**Review characteristics** *(databases, search date)*	**Characteristics of included studies** (number of studies, design, country, setting)	**Patient characteristics**	**Interventions and comparisons**	**Outcome measures and effect size**	**Conclusion**	**Comments**
Effect of physical activity on persons with **ID residing in the community.**	CAB Abstracts, CINAHL, Cochrane Library, ERIC, Medline, Nursing/Academic Edition, PsycINFO, Scopus, SPORT-discus, and Web of Science, August 2012.	N=6, RCT, non-RCT and uncontrolled trials, USA, UK, residing in the community.	Age range 18 to 71. Persons with mild to severe **ID**.	Interventions:<br>- Home exercise program.<br>- Individualized interventions.<br>- Health education.<br>Controls:<br>- NR.	PA:<br>- Increase in mean frequency of self-reported PA.<br>- Increased percentage of time in light intensity PA.<br>- Decrease in sedentary time.	The home exercise and health education interventions improved PA in persons with ID.	+ PA
**Author, publication year: Jansen et al. 2015**<br>**Review aim: Effects of interventions on physical activity in nursing home residents**							
**Review aim**	**Review characteristics** *(databases, search date)*	**Characteristics of included studies** (number of studies, design, country, setting)	**Patient characteristics**	**Interventions and comparisons**	**Outcome measures and effect size**	**Conclusion**	**Comments**
Effect of interventions on	CINAHL, Cochrane Library, DissOnline,	N=8, RCT, Nursing homes,	Mean sample size N=129,	Interventions:<br>- Exercises such as	PA: measured with technical devises, motion sensors, or	There is a positive effect of the interventions on	+PA

physical activity in **nursing home residents.**	Psycarticles CC Med, PsycInfo, Psyndex, Pubmed, and Web of Knowledge. January 2015.	mostly western countries.	Mean age between 75 and 89.	resistance training, chair-based exercises, resistance training etc.<br><br>Controls:<br>- Usual care.<br>- Waiting control.	questionnaires.<br>- 6/8 studies reported significant between-group difference favouring the intervention.<br>- 2/8 studies reported large effect of Cohens d >0.8.<br>- 2/8 found no difference in PA.	the physical activity of the residents.	
**Author, publication year: Temple et al. 2017**<br>**Review aim: Interventions to promote physical activity for adults with intellectual disabilities**							
**Review aim**	**Review characteristics** *(databases, search date)*	**Characteristics of included studies** (number of studies, design, country, setting)	**Patient characteristics**	**Interventions and comparisons**	**Outcome measures and effect size**	**Conclusion**	**Comments**
Study interventions to promote physical activity for adults with **intellectual disabilities.**	CENTRAL, CINAHL, Health Source: Nursing/Academic Edition, Medline, PsycARTICLES, PsycINFO, and SPORTDiscus.January 2015.	N=3, RCT and non-experimental. USA, Sweden, service agency, supported and independent living, clinical research centre.	N=752. Mean age between 18 and 65. Adults with **intellectual disability.**	Interventions:<br>- Physical activity programs with some education.<br><br>Controls:<br>- Not applicable or NR.	PA:<br>- Increase in PA and duration after the intervention.<br><br>- Increase of number steps per day after intervention.<br><br>- 1 study found no improvement in PA.	There is a shortage of evidence to support the effect of interventions to improve PA in persons with intellectual disability. The available evidence shows improvement in steps and duration of PA.	+ PA
**Author, publication year: Wylie et al. 2023**<br>**Review aim: Increasing physical activity levels in care homes for older people: a quantitative scoping review of intervention studies to guide future research**							
**Review aim**	**Review characteristics** *(databases, search date)*	**Characteristics of included studies** (number of studies, design, country, setting)	**Patient characteristics**	**Interventions and comparisons**	**Outcome measures and effect size**	**Conclusion**	**Comments**
Study the effect of interventions	Allied and Complementary	N=19, RCT, quasi-experimental	N= 2445, mean age 85	Interventions:<br>- Multicomponent	PA:<br>- 12/19 studies report a	There is evidence that PA interventions	+PA

to increase PA in **care homes residents.**	Medicine, CINAHL, Embase, Medline, PsychINFO, December 2021.	studies, USA, Netherlands, Spain, China, Scandinavia, Poland, Belgium, Portugal, Australia, elderly care homes.	years, no diseases reported.	exercise.<br>- Home interventions to encourage PA.<br>- Personal activity plan.<br>- Games.<br><br>Controls:<br>- Usual care.	significant improvement of PA measured using accelerometer, survey, active wear armband counting steps.	improve the PA in persons living in care homes.	

Abbreviations: + = significant effect; - = no significant effect; * = pooled effect size; AD = Alzheimer's Disease; ADL = active daily living; BI = Barthel index; ID = intellectual disability; KI = Katz index; LTCF = long term care facility; MD = mean difference; N = number of studies; NR = not reported; PA = physical activity RCT = randomized controlled trial.

Table S2: Data extraction of included studies on technology (n=8; module 'Technology').

**Author, publication year: Agbangla, 2022**<br>**Review aim: To summarize the effects of Snacktivity, giant games and immersive virtual reality exercise among older adults living in nursing homes and long-term care facilities**						
**Review characteristics** *(databases, search date)*	**Characteristics of included studies** (number of studies, design, country, setting)	**Patient characteristics**	**Interventions and comparisons**	**Outcome measures and effect size**	**Conclusion**	**Comments**
Databases: PubMed, PsycINFO, Web of Science.<br><br>Search date > 7 July 2022.	5 studies included 88 participants.<br><br>Design: Quasi-experimental (n=2), within-subject with pre- and post measures (n=1), prospective crossover proof of concept study (n=1), pilot. interventional study (n=1).	Older adults living in nursing homes.	Giant games (n=2): One month of 30-60min per session.<br><br>Immersive virtual reality (n=3): Lasted between 1 hour and 12 weeks. 20 or 30 minutes per session.	Giant games:<br>- Increase in number of steps per day, energy expenditure, quality of life, balance, gait, and ankle strength after 3 months (n=1).<br>Results were confirmed on Tinetti scores, TUG, quality of life, knee extensor isometric strength, grip strength, symmetry of steps and intrinsic motivation (n=1).<br><br>Virtual reality interventions:<br>- Improvement of physical performance compared to exercise without VR (n=1). Cycling was also preferred by participants	Synthesis of the studies included in the current review suggests that new physical practices such as immersive VR, giant games and SnacktivityTM could facilitate the promotion of physical activity. These new physical practices may increase not only the motivation and enjoyment of older people, but also to enhance their functional capacities.	1 out of 5 studies were also included in the review of Chu (2022):<br>- Eisapour (2022)<br><br>1 out of 5 studies were also included in the review of Diener (2022):<br>- Loggia (2021)

	Setting: Nursing homes and long-term care facilities.			with the use of VR (n=1).<br>- Positive effects on subjective responses, motion and fitness parameters (n=1)<br>- Improvement of handgrip strength and walking speed (n=1).		
**Author, publication year: Chen, 2023**<br>**Review aim: To review the training focus of exergames and analyse the effectiveness of exergame training on physical, psychological, or cognitive outcomes as compared with usual care and conventional exercises among older adults in long-term care facilities.**						
**Review characteristics** *(databases, search date)*	**Characteristics of included studies** (number of studies, design, country, setting)	**Patient characteristics**	**Interventions and comparisons**	**Outcome measures and effect size**	**Conclusion**	**Comments**
Databases: Cochrane, CINAHL, Embase, MEDLINE, ProQuest, PubMed, Web of Science<br><br>Search date: > 30 April 2022	12 studies included 482 participants.<br><br>Design: Randomized controlled trial (n=12)<br><br>Country: Australia (n=1), Belgium (n=2), Lebanon (n=1), Myanmar (n=1), New Zealand, (n=2), Spain (n=1), Taiwan (n=1), Turkey (n=1), the United States (n=2).<br><br>Setting: Long-term care facilities	Mean age ranged from 70.1 to 89.5 years old. More females than males in most studies (n=10). Most participants were well functioning (n=7). Others were pre-fail or frail older adults (n=3) or older adults with cognitive impairments (n=2).	Interventions ranged from 3 weeks to 15 weeks. Frequency of session was 2-3 times a week, lasting from 18 min to 60 min.<br><br>The Wii (n=4), Xbox Kinect (n=4), Play Station (n=1) and other studies (n=3) were used.	Physical:<br>- Compared to conventional exercises: significant improvements in balance (SMD 0.49, 95% CI 0.20−0.78, $p < 0.001$).<br><br>Psychological:<br>- Compared to usual care: Significant improvements in balance self-efficacy (SMD 1.04, 95% CI 0.47−1.61, $p < 0.001$).<br><br>Cognitive:<br>- Compared to usual care: Significant improvements in cognition (SMD 0.90, 95% CI 0.61−1.19, $p < 0.001$).<br><br>The remaining outcomes showed no significant improvements: mobility, strength, flexibility, quality of life, activities of daily living.	A total of 12 studies examined the effects of exergames on outcomes of balance, mobility, strength, flexibility, cognition, balance self-efficacy, quality of life, and activities of daily living. Results of the 12 studies included in the meta-analysis indicated that the intervention groups showed significantly improved cognition and balance self-efficacy as compared with the control groups receiving usual care. As compared with the control groups receiving conventional exercises, the intervention groups showed significantly improved balance.	3 out of 12 trials were also included in the review of Chu (2022):<br>- Delbroek (2017)<br>- Mugueta-Aguinaga (2017)<br>- Taylor (2018)<br><br>5 out of 12 trials were also included in the review of Diener (2022):<br>- Delbroek (2017)<br>- Fakhro (2020)<br>- Fu (2015)<br>- Taylor (2018)<br>- Yesilyaprak (2016)<br><br>4 out of 12 trials were also included in the review of Kukkohovi (2022):<br>- Delbroek (2017)

						- Fakhro (2020)<br>- Mugueta-Aguinaga (2017)<br>- Padala (2012)<br><br>2 out of 12 trials were also included in the review of Swinnen (2022):<br>- Padala (2012)<br>- Taylor (2018)
**Author, publication year: Chu, 2022**<br>**Review aim: To summarize the effects of exergaming interventions on physical, cognitive, and quality of life outcomes of older adults living in long-term care settings.**						
**Review characteristics** *(databases, search date)*	**Characteristics of included studies** (number of studies, design, country, setting)	**Patient characteristics**	**Interventions and comparisons**	**Outcome measures and effect size**	**Conclusion**	**Comments**
Databases: CINAHL, PubMed, Web of Science, PsycINFO, ScienceDirect, Cochrane.<br><br>Search date: < July 2020.	21 studies included 657 older adults.<br><br>Design: Randomized control trials (n=9), nonrandomized control studies (n=2), before-and-after studies (n=4), quasi-experimental studies (n=5), interrupted time series study (n=1).<br><br>Country: North America (n=3), Europa (n=10), Oceania (n=3), South	Older adults (>65 years) living in residential long-term care. 275 of 657 older adults were controls. The majority of studies had a higher proportion of women compared to men. The average age ranged from 70.1 to 90.4 years.	Exergaming.<br><br>Intervention sessions occurred at least twice a week (76% of the studies), mostly more than 4 weeks (62%). Length of exergaming sessions was variable across studies, with the most begin approximately 30-minute sessions.<br><br>81% used commercially available hardware (Nintendo Wii / Xbox Kinect). All the	Physical outcomes (n=20):<br>- Medium to large effect size on mobility and endurance (d =0.55 – 1.01) (n=8).<br>- Mixed results on balance (n=8).<br>- Significant improvements in gait (n=3).<br>- Significant improvements in reaction time (n=2).<br>- Significant improvements in physical well-being and PA levels (n=5).<br><br>Cognitive outcomes (n=5):<br>-Two studies found better cognitive outcomes. Three studies found no significant differences.<br><br>Quality of life outcomes (n=6):<br>- One study showed significant improvements for psychological QoL.<br>- One study found significant	Exergame interventions were associated with preliminary benefits relative to control conditions on standardized measures of physical outcomes. Especially reaction time, physical wellbeing and physical activity highly improved.<br>No effects were found for cognitive and quality of life outcomes.	The majority (66%, n = 14/21) of studies were associated with a serious risk of bias due to lack of randomization, uncontrolled study designs, and lack of blinding due to the nature of the intervention.

	America (n=1), Asia (n=4).<br><br>Setting: Long-term care.		intervention/exergaming sessions were supervised by either a member of the research staff (n=7),<br>a physical therapist (n=5),<br>an exercise therapist (n=3),<br>nursing staff (n=2), or volunteer (n=1).	improvement on vitality, mental health-related QoL and physical functioning.<br>- Four studies found no improvements on QoL scales.		
**Author, publication year: Diener, 2022**<br>**Review aim: To provide an overview of the effectiveness, acceptability, and feasibility of e- and m-health interventions aimed at promoting physical activity and preventing falls in nursing homes.**						
**Review characteristics** *(databases, search date)*	**Characteristics of included studies** (number of studies, design, country, setting)	**Patient characteristics**	**Interventions and comparisons**	**Outcome measures and effect size**	**Conclusion**	**Comments**
Databases: PubMed, Scopus, SPORTDiscuss, Web of Science.<br><br>Search date: > 15 November 2021.	28 studies included.<br><br>Design: Randomized controlled trials (n=12), cross-over randomized controlled trials (n=2), cluster-randomized controlled trials (n=2), controlled trials (n=4), pre/post intervention studies (n=4), other (n=4).<br><br>Country: Europa (n=10), Asia (n=10), Australia/New Zealand (n=4), Africa (n=1), North America (n=1).	Studies exclusively focusing on nursing home residents were included.<br><br>The mean age ranged from 66.9 to 88.9 years.	E-health and m-health interventions:<br>- 24 studies on digital exergaming, of which 17 used commercially available gaming (Nintendo Wii and Xbox with Kinect).<br>- 4 studies on e-health interventions.<br>- no m-health interventions.	Physical activity (n=6):<br>- Two studies reported significant improvements on overall physical activity, measured with questionnaires.<br>- One study reported no significant differences measured with body-worn sensors.<br>- Three studies reported activity during the sessions, of which two studies scored higher physical activity.<br><br>Falls (n=21):<br>- Three studies assessed number of falls, of which two found significant decreases in falls.<br>- 19 studies assessed balance tests. Nine studies used the Timed Up and Go Test (TUG) which all showed significant improvements. Even compared to balance and strength training (n=2) or bike	Data synthesis indicates that exergaming may be effective in reducing the number of falls and fall risk in NH residents. Based on the included studies, the duration of the intervention does not seem to influence effectiveness, while the results indicate that interventions with three or more sessions per week tend to be more effective.<br>Conversely, due to the limited number of studies and heterogenous results, the impact of exergaming on PA levels of NH	Residents with advanced physical and/or cognitive impairments were excluded in most studies.<br><br>Four e-health interventions were also based on exergaming (e.g. cybercycling).<br><br>10 out of 28 trials were also included in the review of Chu (2022):<br>-Cicek (2020)<br>-Delbroek (2017)<br>-Keogh (2012)

	Setting: Nursing homes / Long-term care facilities.			ergometer and treadmill training (n=1). No differences on balance were found compared to conventional balance training (n=2).<br>- Two studies on fall risk also showed significant improvements.<br>- One study used sensors to measure balance which showed that total time, turn-to-sit transition, and the step-time before the turn significantly decreased. Sit-to-stand transition nor turn duration did not change.<br>- Six studies showed heterogeneous results on fall risk. Two found significant improvement compared to usual care, two did not. Three studies reported significant improvement post-measured.<br>- Also heterogeneous results for other balance tests. Five studies reported significant improvements for exergaming post intervention, two did not. Two found significant improvements compared to control or sham interventions, two did not. Compared to conventional training, two studies found significant improvements, and one did not.<br><br>Secondary outcomes: Heterogeneous results were found for falls efficacy, quality of life, neuropsychiatric symptoms, cognitive function, strength parameters, static balance, mobility scores, walking speed, foot placement, aerobic capacity.	residents seems to be unclear. Regarding secondary outcomes, exergaming demonstrated significant improvements, although the results were not consistent for all outcomes. The two studies on cybercycling showed contrasting results in terms of an increased cycling distance compared to conventional stationary cycling. One study with a VR-based horticultural therapy program found that the intervention was highly accepted by NH residents. The provision of a falls prevention program via videoconferencing did not lead to a significant reduction in the number of falls. No conclusion can be drawn about the effects of exergaming and other e-health interventions on PA, as data is scarce.	-Keogh (2014)<br>-Monteiro-Junior (2017)<br>-Pichierri (2012)<br>-Rogan (2016)<br>-Taylor (2018)<br>-Wu (2019)<br>-Zahedian-Nasab (2021)
**Author, publication year: Kukkohovi, 2023**<br>**Review aim: To identify, critically appraise, and synthesize evidence about the effectiveness of playing digital games on older people's physical, psychological, and social functioning and physical and social activity in long-term care facilities**						

**Review characteristics** *(databases, search date)*	**Characteristics of included studies** (number of studies, design, country, setting)	**Patient characteristics**	**Interventions and comparisons**	**Outcome measures and effect size**	**Conclusion**	**Comments**
Databases: PubMed, Web of Science, CINAHL, Scopus, Cochrane.<br><br>Search date: > November 2021.	15 studies included 674 older adults.<br><br>Design: Randomized controlled trials (n=6) and quasi-experimental studies (n=9).<br><br>Country: Belgium (n=2), the Netherlands (n=1), Turkey (n=1), The United Kingdom (n=1), Lebanon (n=1), Taiwan (n=1), Singapore (n=1), Iran (n=1), USA (n=1), Brazil (n=2), South Africa (n=1), Australia (n=1).<br><br>Setting: Long-term care facilities.	Older people without specific diseases excluding memory disorders living in long-term care.<br><br>Mean age; 66.5 – 87.5 years.<br><br>Disorders:<br>- Cognitive disorders/Alzheimer's disease (n=4).<br>- Poor balance (n=1)<br>- (risk of) frailty syndrome (n=2).	All digital games were exergames.<br>- Nintendo Wii (n=6).<br>- Xbox-360 Kinect (n=4).<br>- Kinect motion sensors (n=3).<br>- A force platform (n=2).<br><br>Interventions lasted from 3 to 12 weeks, with 8 and 1 2weeks being most popular. In most of the study participants played three times a week. Sessions lasted from 15 min to 1.5h. Sessions were played individual (n=6) in pairs (n=1) in group (n=3) or individual or in a group (n=1). In four studies that was unknown.	Physical functioning: 8 of 10 studies showed positive changes. Statistically significant large effect [number of studies (N) = 6, standardized mean difference (SMD) = 0.97, p = 0.001].<br><br>Psychological functioning: 3 of 4 studies reported positive change in depression scale. The meta-analysis on RCTs showed no significant difference (SMD = 0.52, 95% CI [− 0.59, 1.63], p = 0.35). The same for quasi-experimental studies: (SMD = 1.46, 95% CI [− 0.46,3.38], p = 0.14).<br><br>Social functioning: 3 of 5 studies showed positive results, of which one was not significant. Medium effect (N = 5, SMD = 0.74, p = 0.016).<br><br>Self-assessed physical activity: 4 of 4 studies reported increase, of which one was not significant. Statistically significant large effect (N = 3, SMD = 1.20, p < 0.001).	Based on the results of this review, exergaming has a positive effect on the physical functioning of long-term care residents measured by TUG or SPPB (=physical functioning).<br><br>Playing digital exergames seems to be effective in promoting older people's physical and social functioning and in increasing their physical activity in a long-term care environment. Social activity was not measured in any study. The effects in terms of psychological functioning are not completely clear, but playing exergames has been able to have a positive effect on the reduction of depressive symptoms.	5 out of 15 trials were also included in the review of Chu (2022):<br>- Cicek (2020)<br>- Delbroek (2017)<br>- Janssen (2013)<br>- Keogh (2014)<br>- Mugueta-Aguinaga (2017)<br><br>8 out of 15 trials were also included in the review of Diener (2022):<br>- Babadi and Daneshmandi (2021)<br>- Cicek (2020)<br>- Delbroek (2017)<br>- Fakhro (2020)<br>- Janssen (2013)<br>- Keogh (2014)<br>- Ramnath (2021)<br>- Rica (2020)
**Author, publication year: Lancioni, 2022**<br>**Review aim: To provide a comprehensive picture of intervention programs using stimulation-regulating technologies to promote forms of physical activity in people with intellectual and multiple disabilities.**						

**Review characteristics** *(databases, search date)*	**Characteristics of included studies** (number of studies, design, country, setting)	**Patient characteristics**	**Interventions and comparisons**	**Outcome measures and effect size**	**Conclusion**	**Comments**
Databases: PubMed, Web of Science, PsycINFO, ERIC, CINAHL, Google Scholar.<br><br>Search date: 2010 – 2021.	42 studies were included with in total 465 participants.<br><br>Design: Not reported.<br><br>Country: Italy (n=15), Taiwan (n=14), United States (n=5), Chile (n=1), Egypt (n=1), France (n=1), Hong Kong (n=1), Israel (n=1), New Zealand (n=1), Portugal (n=1), the Netherlands (n=1).<br><br>Setting: Not reported.	Individuals with intellectual disability or a combination of intellectual disability with additional disorders, such as sensory and motor impairments.	Two groups of interventions:<br><br>1. Technology-regulated delivery of preferred stimulation contingent (n=27), e.g. use of sensors, balance boards or dance boards.<br><br>2. Video games and related auditory and visual stimulation (n=15), e.g. virtual reality or Wii Fit.	Specific responses for both response-contingent stimulations as well as exergames were reported. The reviews provide a narrative description of all studies.<br><br>Two studies showed an increase in physical activity level.<br>The studies reported lots of positive effects on physical outcomes such as walking, heart rate, body movements, stretching, ambulation responses, coordination, muscle strength, motor proficiency, running, feet lifting. Also, three studies reported positive effects on balance. Cognitive functioning was improved in one study. Four studies reported a positive effect on indices of happiness and three studies on satisfaction. Positive participation, self-injurious behaviour, enjoyment, happiness and well-being was also improved in one study each.	All studies based on the use of response-contingent stimulation reported positive outcomes.<br><br>Most of the studies based on exergames also showed positive outcomes. One study was partially positive and two studies were mainly positive. One study showed inconclusive effects.	The review included small study designs.<br><br>The review reported very specific responses.
**Author, publication year: Li, 2023**<br>**Review aim: to systematically summarize randomized controlled trials that examine the effects of VR-based exercise on different types of health-related fitness (HRF) and skill-related fitness (SRF) in individuals with intellectual disability**						
**Review characteristics** *(databases, search date)*	**Characteristics of included studies** (number of studies, design, country, setting)	**Patient characteristics**	**Interventions and comparisons**	**Outcome measures and effect size**	**Conclusion**	**Comments**

<table>
<tr>
<td>Databases: Embase, PubMed, Web of Science, Cochrane, SPORTDiscuss, Scopus.

Search date: > August 15, 2022.</td>
<td>13 studies included 803 participants with intellectual disability.

Design: Randomized controlled trials.

Country: Not reported.

Setting: Not reported.</td>
<td>Persons with intellectual disability.

More males (n=354) than females (n=217) participated. Ages ranged from 5 to 77.

Eight studies involved children and adolescents,
five studies included adults.

Severity levels ranged from mild to moderate intellectual disability, reported by 8 studies.</td>
<td>Virtual reality-based exercise as primary intervention:
- Nintendo Wii (n=8).
- Xbox kinect (n=2).
- Sony's playstation (n=1).
- VR system (n=1).
- SeeMe virtual games (n=1).
- VR-training combined with treadmill training or traditional therapy (n=2).

Control group:
- Blank control group (n=9).
- Physical education (n=2).
- Standard occupational therapy (n=1).
Traditional physical therapy (n=1).

Duration of the intervention ranged from 4 weeks to 1 year. Weekly frequency between two and four times. Session duration ranged from 15 to 60 minutes.</td>
<td>- Muscular fitness: Generally reported significant improvements (d = 0.05–2.12; proportion of effect = 5/6).
- Cardiorespiratory fitness: Effective improvement (d = 0.12–0.67; proportion of effect = 5/5).
- Flexibility: Heterogeneous results: One study reported significant improvement (d = 0.81; proportion of effect = 1/2), and the other did not report significant improvement.
- Balance: Mostly positive effects (d = 0.23–1.65; proportion of effect = 6/7).
- Coordination: Partly improved (d = 0.19–0.48; proportion of effect = 2/3).
- Speed and agility: Effective improvement (d = 0.13–0.46; proportion of effect = 4/4).
- Overall motor proficiency: Partly improved (d = 1.08; proportion of effect = 1/3). Two studies reported no improvement.</td>
<td>The study provided preliminary evidence that VR-based exercise could improve muscular fitness, cardiorespiratory fitness, balance, and speed and agility in individuals with ID.
The outcomes flexibility, coordination, and overall motor proficiency showed less conclusive or limited positive evidence.</td>
<td>Eight studies involved children and adolescents.

9 out of 13 studies were also included in the review of Lancioni (2022):
- Gomez Alvarez (2018)
- Hsu (2016)
- Lau (2020)
- Lin (2012)
- Lotan (2010)
- Perrot (2021)
- Rahman (2010)
- Silva (2017)
- Wuang (2011)</td>
</tr>
<tr>
<td colspan="7">Author, publication year: Swinnen, 2022
Review aim: To investigate the efficacy of exergames for people with major neurocognitive disorder.</td>
</tr>
<tr>
<td>Review characteristics (databases, search date)</td>
<td>Characteristics of included studies (number of studies,</td>
<td>Patient characteristics</td>
<td>Interventions and comparisons</td>
<td>Outcome measures and effect size</td>
<td>Conclusion</td>
<td>Comments</td>
</tr>
</table>

	design, country, setting)					
Databases: PubMed, EMBASE, PEDro.<br><br>Search date: > October 2019.	8 studies included 671 participants.<br><br>Design: Randomised controlled trials (n=6), pilot randomised trial (n=1), and clinical controlled trials (n=1).<br><br>Country: Not reported.<br><br>Setting: Participant in the studies were community-dwellng (n=7), in long-term care facilities (n=6), assisted living facilities (n=1), or a combination (n=2).	Both adults with major neurocognitive disorders as well as healthy older adults were included.<br>Age ranged from 49 to 95 years old.	Exergames<br>- Session planning varied.<br>- Most studies used commercial available exergames (Xbox (n=5), Nintendo Wii (n=6) or a combination (n=1). One study used Physiomat, and two others used Bike Labyrinth.<br>- Group setting (n=4) and individual (n=4).	Physical outcomes:<br>- 6 studies found improvements on gait, mobility and balance.<br><br>Cognitive outcomes:<br>- One study showed significant improvements on global cognition, episodic memory, and executive functioning.<br>- One study showed no significant effects for executive functioning, episodic memory, working memory.<br>- Three studies found no effects on cognitive functioning.<br><br>Mental outcomes:<br>- One study found improvement in activities of daily living. Four studies did not.<br>- One study found no effects on depressive symptoms.<br>- One study found increase in quality of life (which was also found for the control walking group). Three other studies found no improvements.<br>- One study found significant effect on balance and falls efficacy.<br><br>Study attrition was high in all studies: Adherence ranged from 69% to 100% in intervention groups (mean = 84,4%) and 78% to 100% in control groups (mean = 85,8%).	Positive effects of exergames in people with major neurocognitive disorder were most prominent in several physical outcomes, such as gait, mobility and balance. The preliminary results also<br>show promising effects on cognitive functioning, activities of daily living performance, fear of falls, quality of life and mood.<br><br>Finally, and of importance for clinical practice, it remains inconclusive whether the exergame intervention is superior of inferior compared to a regular exercise intervention.	1 out of 8 trials were also included in the review of Kukkohovi (2023):<br>- Padala (2012)<br><br>1 out of 8 trials were also included in the review of Chu (2022):<br>- Taylor (2018)<br><br>1 out of 8 trials were also included in the review of Diener (2022):<br>- Taylor (2018)

Table S3: Data extraction of included studies on motivation (n=11, modules 'Motivating clients', 'Encouraging informal caregivers' and 'Organisation of care').

**Author, publication year: Aitchison, 2022**<br>**Review aim: To explore and understand the sport experiences and perceived health benefits of sport across four different populations: children and adolescents, adults, elite athletes and veterans with a disability**						
**Review characteristics** *(databases, search date)*	**Characteristics of included studies** (number of studies, design, country, setting)	**Participant characteristics**	**Results**	**Influencing factors**	**Conclusion**	**Comments**
Systematic review.<br><br>Databases: Medline, EMBASE, PsychINFO, Web of Science, CINAHL Plus, SportDiscus, and grey literature.<br><br>Search date > 29 February 2020.	39 studies included, of which 22 were on adults.<br><br>Design: Qualitative studies (n=34), quantitative studies (n=2), mixed-methods (n=3).<br><br>Country: Not reported.<br><br>Setting: Not reported.	Individuals with a physical, visual or intellectual disability who participated in sport competitively or recreationally were included. All ages. >6 months participating in sports or sport camps.	Four themes were described for adults. Sports had a positive effect on:<br>1. Liberation<br>2. Inclusion<br>3. Breaking barriers<br>4. Life-changing	Seven studies described barriers and facilitators to sport participation:<br>- Surrounding accessibility<br>- Specialist equipment<br>- Volunteers<br>- Guide runners<br>- Disability itself	Sport is a beneficial experience for individuals with a disability. Positive aspects should be promoted more to encourage sport participation.	

**Author, publication year: Anderiesen, 2014**<br>**Review aim: Investigate the influence that the environment has on level of physical activity**						
**Review characteristics** *(databases, search date)*	**Characteristics of included studies** (number of studies, design, country, setting)	**Participant characteristics**	**Results**	**Influencing factors**	**Conclusion**	**Comments**
Systematic review<br><br>Databases: PubMed, PsycINFO,	24 studies included.<br><br>Design: Randomized controlled trials (n=7), pseudorandomized	Older persons with dementia living in nursing homes.	1. Ambient features (n=9)<br>- Effects of lights are associated with improved sleep-wake rhythms, which are determined by	1. Ambient features (n=9)<br>- Bright light throughout the day.<br>- Timed bright light.<br>- Music.	Positive results on the residents' levels of physical activity were found for music, a homelike	Activities of daily living, such as oral care, were also seen as

EMBASE, CINAHL, Cochrane.<br><br>Search date: January 1993 – December 2022.	controlled trial (n=4), non-randomised experimental study (n=2), cross-sectional (n=2), pre-test/post-test (n=2), five single arm (n=1), two-single arm (n=1), case series (n=3).<br><br>Country: Not reported.<br><br>Setting: Nursing homes, dementia care units, small living facilities.		monitoring physical activity.<br>- Mixed results on bright light and timed light.<br>- Music positively influences activities of daily living.<br><br>2. Interior design features (n=7)<br>- Small improvement in activity adherence for homelike environment<br>- Mixed results on the effects of a multisensory environment.<br>- Functional modifications, such as verbal cueing, improved activities of daily living.<br><br>3. Architectural features (n=8)<br>- Predominantly positive results for small-scale group living concepts.<br>- Mixed results on footprint planning.	2. Interior design features (n=7)<br>- Homelike environment.<br>- Multisensory environment (Snoezelen).<br><br>3. Architectural features (n=8)<br>- Small-group living concepts.	environment and functional modifications. Predominantly positive results were also found for the small-scale group living concepts. Mixed results were found for bright or timed light, the multisensory environment and differences in the building footprint.	physical activity.

**Author, publication year: Benjamin, 2014**<br>**Review aim: To present an overview of the literature related to the barriers to physical activity and restorative care withing long-term care settings.**						
**Review characteristics** *(databases, search date)*	**Characteristics of included studies** (number of studies, design, country, setting)	**Participant characteristics**	**Results**	**Influencing factors**	**Conclusion**	**Comments**
Review of the literature.<br><br>Databases: MEDLINE,	7 studies included.<br><br>Design: Qualitative studies (n=7).	Residents living in long-term care.	1. Resident-related barriers<br>- Poor health status (n=7) (e.g. physical limitations or lack of energy).<br>- Use of sedative medications	Barriers experienced by residents:<br>- Poor health status of residents.<br>- Fear of falling and/or injury.<br>- Past history of sedentary lifestyle (physical activity is not a habit / now it is time to rest).	Barriers occurred at resident (e.g., health status), environmental (e.g., lack of space for physical	Focus on physical activity and restorative care

CINAHL, AgeLine, SPORTDiscuss.<br><br>Search date: 2002 – 2013.	Country: United states (n=4), Canada (n=1), Germany (n=1), Australia (n=1), Taiwan (n=1).<br><br>Setting: Nursing home, long term care home, home for the aged, assisted living, residential care.		(n=3).<br>- Anxiety and agitation (n=1).<br>- Fear of falling and/or injury (n=5).<br>- Past history of sedentary lifestyle (n=2) (physical activity is not a habit / now it is time to rest).<br>- Lack of knowledge and motivation ("too old to exercise") (n=2).<br><br>2. Organisational barriers<br>- Funding limitations and staffing constraints (n=1).<br>- Lack of time to incorporate physical activity into resident's daily routine (n=2).<br>- Timing of classes (n=1) (e.g. morning fatigue, toilet routine, medication effects).<br>- Competing demands and schedules (n=2).<br>- Lack of communication among staff (n=1).<br>- Lack of support and encouragement from staff (n=3).<br>- Exercises classes did not fit: not challenging enough, boring or fear they would not be able to "keep up" (n=3).<br>- Institutional routines (e.g. bath and mealtime) (n=2).<br>- Doctor did not recommend to exercise (n=1).<br><br>3. Environmental barriers<br>- Limited living space (n=4).	- Competing demands and schedules.<br>- Lack of support and encouragement from staff.<br>- Exercise classes did not fit their preferences.<br>- Institutional routines (e.g. bath time).<br>- Lack of knowledge and motivation ("too old to exercise").<br>- Timing of classes.<br>- Doctor did not recommend to exercise.<br><br>Barriers experienced by staff:<br>- Poor health status of residents.<br>- Use of sedative medications.<br>- Anxiety and agitation.<br>- Fear of falling and/or injury.<br>- Funding limitations and staffing constraints.<br>- Lack of time to incorporate physical activity into resident's daily.<br>- Lack of communication among staff.<br>- Institutional routines (e.g. mealtime).<br><br>Barriers experienced by significant others:<br>No specific barriers were mentioned for significant others (although barriers might overlap the above).	activity), and organisational (e.g., staffing and funding constraints) levels. These barriers intersect to adversely affect the physical activity of older people living in long-term care.	(=restoring or maintaining a resident's physical function).

			- Lack of designated areas for exercise / lack of space for equipment (n=1).<br>- Lack of seating in the corridors to take rest breaks<br>- Lack of exercise equipment (n=1).<br>- Uneven surfaces in the outdoor spaces (n=1).			
**Author, publication year: Bossink (2017)**<br>**Review aim: Identify barriers and facilitators of physical activity in people with intellectual disability, examine differences between levels of intellectual disability, and between various stakeholder groups.**						
**Review characteristics**<br>*(databases, search date)*	**Characteristics of included studies**<br>(number of studies, design, country, setting)	**Participant characteristics**	**Results**	**Influencing factors**	**Conclusion**	**Comments**
Systematic review.<br><br>Databases: MEDLINE, ERIC, PsycINFO.<br><br>Search date: January 1990 – September 2015.	24 studies included 684 participants: 264 with ID, 221 direct support professionals, 33 indirect support professionals, and 166 parents.<br><br>Design: Qualitative studies (n=20) and quantitative studies (n=4).<br><br>Country: USA (n=5), United Kingdom (n=8), Ireland (n=1), Italy (n=1), Australia (n=4), Sweden (n=1), the Netherlands (n=1), Canada (n=1), China (n=1).<br><br>Setting: Residential care (n=6), home (n=10), supported work site (n=2),	- People with mild to moderate ID (n=13).<br>- People with severe to profound ID (n=2).<br>- People with ID level not reported (n=10).<br>- Direct support professionals (e.g. group-home supervisors, volunteers) (n=15).<br>- Parents (n=11).<br>- Indirect	14 personal factors were identified: Fear, financial resources, health issues, motivation, preferences, intellectual ability/disability, physical abilities/disabilities, age, physical (dis)comfort, challenging behaviour, behavioural skills, routine, social interaction, being rewarded.<br><br>23 environmental factors were identified: lack of financial support, limited option for physical activity, anxiety (staff and parents), time constraints (parents), competitive component, staffing level, transport,	FACILITATORS<br>*Personal:*<br>- Social interaction.<br>- Being rewarded or praised for participation.<br>- Social engagement with peers, friends, or a team.<br>- Routine.<br>- Physical and intellectual ability.<br>- Feeling good and energetic.<br>- Younger age.<br><br>*Environmental:*<br>- Activities with an element of fun.<br>- One-to-one programs to meet individual needs.<br>- Having a pet.<br>- Regular nature of programs.<br>- Support from a research team.<br>- Staff interest and support.	This systematic review of a sample of 24 studies identifies 37 factors that impede or facilitate physical activity participation in people with ID. The quality ratings of the studies varied, particularly for the qualitative studies. The results indicate that full or partial barriers are reported more frequently than facilitators are. The most frequently reported barriers were related to	

	day-care centers (n=1), group home (n=2), special educational needs schools (n=1).	support professionals (e.g. service managers, program coordinators) (n=4).	weather/season, community support, staff expertise, societal influences, policy guidelines, lack of inclusion, family support, geographical location and environment, work routines, adapted and accessible activities, staff interest, regular nature of physical activity, activity with fun component, one-to-one nature, external research team, having a pet.<br><br>Level of ID:<br>Some (n = 6) of the studies noted that the level of ID plays a crucial role with regard to participation in physical activity. Greater severity of ID and the related need for supervision were described as limiting physical activity (n=6). level of ID is a determinant of the extent to which an intervention could be delivered, as well as the manner in which it could be delivered (n=1).<br><br>Differences between barriers and facilitator of the stakeholder groups were found.	- Presence of good support, warm working climate, encouragement.<br>- Existence of policy guidelines.<br>- Family support.<br>- Geographical location.<br><br>BARRIERS<br>*Personal:*<br>- Fear (e.g. of falling).<br>- Routine.<br>- Health status (e.g. overweight, illness, ear problems, heart conditions).<br>- Motivations and preferences of people with ID.<br>- Physical disabilities, physical discomfort, lower intellectual functioning, ageing.<br>- Challenging behaviour.<br><br>*Environmental:*<br>- Lack of financial support.<br>- Limited options.<br>- Anxiety (staff and parents).<br>- Time constraints (parents).<br>- Competitive activities.<br>- Lack of adapted and accessible activities.<br>- Staff interest and support.<br>- Irregular nature of programs.<br>- Low staffing levels.<br>- Transport difficulties.<br>- Lack of community support (e.g. discontinued classes, lack of acceptance).<br>- Lack of clear policy guidelines in local service agencies.<br>- Weather constraints.<br>- Staff expertise.<br>- Negative social influences.	health issues, motivation and preferences, financial support, staffing levels, and transportation.	

				- Lack of inclusion.<br>- Work routines.		
**Author, publication year: Douma (2017)**<br>**Review aim: Review setting-related factors (including social and physical environment) that may contribute to the amount of older adult's physical inactivity in a wide range of residential care settings.**						
**Review characteristics**<br>*(databases, search date)*	**Characteristics of included studies**<br>(number of studies, design, country, setting)	**Participant characteristics**	**Results**	**Influencing factors**	**Conclusion**	**Comments**
Literature review.<br><br>Databases: PubMed, PsychINFO, Embase, CINAHL, Cochrane.<br><br>Search date > 16 July 2015.	12 studies included.<br><br>Design: Qualitative (n=6), mixed methods (n=1), quantitative (n=5).<br><br>Country: United states (n=7), Taiwan (n=1), Germany (n=1), Sweden (n=3).<br><br>Setting: Long-term care facilities.	Residents in long-term care facilities, such as nursing homes.	*Influence of the physical environment (n=5):*<br>- person-environment fit is related to more activity (e.g. right height of kitchen shelves).<br>- characteristics of the building layout and interior influence inactivity levels: more office space and distances, improved accessibility features, physical amenities, presence of little more features concerning security (e.g. call buttons).<br>- security, accessibility and comfort increases corridor walking (e.g. handrails, safe carpet, wide corridors, possibility to sit, both long or short walking distances, large elevator dimensions, accessibility of activity areas and restrooms). Barriers: limited walking areas, not many things to see indoors	- Amount of caregivers.<br>- Time that caregivers have available.<br>- Amount and type of care.<br><br>*Facilitators:*<br>- Person-environment fit (e.g. right height of kitchen shelves).<br>- More office space and distances.<br>- Improved accessibility features.<br>- Physical amenities.<br>- Presence of little more features concerning security (e.g. call buttons, handrails, safe carpet).<br>- Accessibility and comfort (e.g. wide corridors, possibility to sit in corridor, accessibility of activity areas).<br><br>*Barriers:*<br>- Limited walking areas.<br>- Not many things to see indoors (artwork, plants etc. in the corridor are appreciated).<br>- Having nothing to do.	Inactivity levels in residential care settings may be reduced by improving several features of the physical environment and with the help of caregivers.	

			(artwork, plants etc in the corridor are appreciated).<br>- indoor facilities not related to more activity.<br><br>*Influence of caregivers on physical inactivity levels of residents (n=5):*<br>- Number of caregivers; more inactivity during weekends compared to weekdays; more inactivity in homes with lower staff levels.<br>- Time that caregivers have available; Less time leads to more inactivity (e.g. less walking).<br>- Amount and type of care: Activating care is stimulating.<br>- Having nothing to do increases inactivity.			
**Author, publication year: Jacinto (2021)**<br>**Review aim: Update knowledge about the perceived barriers of physical activity participation in individuals with intellectual disability**						
**Review characteristics**<br>*(databases, search date)*	**Characteristics of included studies**<br>(number of studies, design, country, setting)	**Participant characteristics**	**Results**	**Influencing factors**	**Conclusion**	**Comments**
Systematic review.<br><br>Databases: PubMed, SPORTDiscus, Web of Science, Scopus.<br><br>Search date: 2016 to May 2021.	5 studies included 181 participants, of which 56 were individuals with intellectual disability.<br><br>Design: Exploratory study (n=2), qualitative study	- Individuals with intellectual disability (Down syndrome included), of any age, gender, ethnicity or	Barriers of physical activity participation:<br><br>*1. Personal:* Characteristics of the disability itself (physical, physiological and psychological); acceptance of inactive lifestyles; aging; health problems; lack of	<u>Barriers:</u><br>*Perception of individuals:*<br>- Personal: Preference for inactive lifestyles, aging, health problems.<br>- Social: Lack of adapted spaces, lack of inclusion, lack of places to practice, lack of adapted PA.<br>- Environmental: Adverse weather	These studies revealed the existence of several perceived barriers to regular PA participation, which were grouped into five	Update of Bossink (2017)

	(n=2), cross-sectional study (n=1).<br><br>Country: Italy (n=1), United Kingdom (n=2), USA (n=1), Australia (n=1).<br><br>Setting: Not reported.	race.<br>- Family members.<br>- Caregivers.	concentration; challenging behaviours.<br>*2. Family members:* Parents' concerns (bullying, among others); acceptance of inactive lifestyles; communication problems with technical caregivers; lack of time to engage in PA with their children.<br>*3. Social:* Acceptance of inactive lifestyles; lack of information on adapted PA; lack of adapted PA programs; lack of inclusive opportunities; lack of technicians specialized in adapted PA; lack of places to practice PA; limitation of human resources; other preferences of the institution providing support services; communication problems between family members and caregivers; social exclusion (stigma and lack of understanding in relation to disability); lack of support; sensory issues (music too loud in training places); difficulties in transportation (high costs, lack of transport).<br>*4. Financial:* Limited financial resources.<br>*5. Environmental:* Climate.	conditions.<br><br>*Perception of family members:*<br>- Social: Lack of specialists in adapted PA, lack of adapted PA programs, lack of inclusive programs, difficulties in transportation, lack of information about adequate and inclusive PA, social exclusion, lack of support, stigma and lack of understanding of disability.<br>- Family members: Parents' concerns, lack of time for parents to engage in PA with children, acceptance of children's inactive lifestyles, communication problems between family members and caregivers.<br>- Financial: Limitation of economic resources.<br><br>*Perception of technical caregivers:*<br>- Personal: Aging, health problems, low concentration capacity, challenging behaviours<br>- Social: Lack of adapted spaces, lack of inclusion, stigma, sensory issues, lack of human resources in institutions, acceptance of inactive lifestyles, other preferences of technical caregivers and institutions, communication problems between family members and caregivers.<br>- Financial: Limitation of financial resources.	main groups: personal (6 topics), family (4 topics), social (13 topics), financial (1 topic) and environmental (1 topic).	
**Author, publication year: Laxton (2023)**<br>**Review aim: Synthesize the multilevel factors related to physical activity among adults with intellectual disabilities in group homes**						

**Review characteristics** *(databases, search date)*	**Characteristics of included studies** (number of studies, design, country, setting)	**Participant characteristics**	**Results**	**Influencing factors**	**Conclusion**	**Comments**
Systematic review<br><br>Databases: Web of Science, MEDLINE, PubMed, PsycINFO, CINAHL.<br><br>Search date > April 2022.	10 studies included.<br><br>Design: Qualitative (n=9), quantitative (n=1).<br><br>Country: United Kingdom (n=3), USA (n=2), Hong Kong (n=1), Norway (n=1), Ireland (n=1), Australia (n=1), Sweden (n=1).<br><br>Setting: Group homes were defined as community residential settings that provided a home-like environment for a smaller group of residents.	Adults (age 18-65) with intellectual disability living in group homes.	- Demographics: Older age, being male, being employed, personal financial constraint.<br><br>- Intrapersonal factors: health and functional status, knowledge, attitude, skills, physical activity behaviour, other behavioural factors.<br><br>- Interpersonal factors: Staff, peers.<br><br>- Environmental factors: Layout, equipment, lifestyle, neighbourhood, local amenities, fitness amenities.<br><br>- Organisational factors: Policy, program offerings, staff education, funding, culture.	<u>Facilitators/positive impact:</u><br>-- DEMOGRAPHIC: Being employed (n=2).<br>-- INTRAPERSONAL:<br>- knowledge: Knowledge of benefits of PA (n=2).<br>- Attitude: Perception of PA activities as being "fun" (n=4), desire to counteract sitting time (n=1).<br>-PA behaviour: Walking (n=4), chores (n=2).<br>- Other behavioural: Receiving rewards (n=5), have a daily routine (n=3), having a fitness membership (n=2).<br>-- INTERPERSONAL:<br>- Staff: Staff's attitude (n=4), staff willingness (n=3), staff commitment (n=5), staff encouragement (n=5), coparticipation (n=2), staff as role models (n=3), staff presentation (n=1).<br>- Peers: Peer's interest in PA (n=3), Being a part of a team/social benefits (n=3).<br>-- ENVIRONMENTAL:<br>- Layout: sufficient space (n=1).<br>- Equipment: Availability of PA equipment (n=1), technology (n=1).<br>- Lifestyle: having a pet (n=1).<br>- Neighbourhood: accessible to sidewalks (n=1).<br>- Local amenities: Nearby parks (n=1), nearby shopping (n=1).<br>- Fitness amenities: community facilities (n=1).<br>-- ORGANISATIONAL:<br>- Policy: PA as part of daily routine (n=3).<br>- Program offerings: Outings (n=2), group	In the qualitative studies reviewed (n = 9), barriers and facilitators relating to PA were discussed at all levels of the ecological model. Intrapersonal factors (e.g., health and function status, knowledge and attitudes about PA), interpersonal factors (e.g., staff attitudes to PA, encouragement for PA, and co-participation in PA), and organisational factors (e.g., program offerings, staff education, and staff–client ratios) were all commonly discussed as influencing PA participation.	

				programming (n=1), diversified PA programs (n=2), day program (n=2).<br>- Staff education: Staff development program (n=1), staff knowledge/creativity (n=1).<br>- Funding: Funding for staff development (n=1), university partnerships (n=1).<br><br><u>Barriers/Negative impact:</u><br>-- DEMOGRAPHIC: Older age (n=4), Being male (n=1), Being employed (n=1), Personal financial constraint (n=2).<br>-- INTRAPERSONAL:<br>- health and functional status: Obesity (n=3), comorbidities (n=4), lack of energy (n=2), behavioural issues (n=1), limited attention (n=1), sensitivity to sounds (n=1), mobility impairment (n=1), resident cognition (n=1), lack of independence (n=1).<br>- knowledge: lack of knowledge of PA effects/misinterpret body signals (n=3).<br>- Attitude: disinterest in PA (n=1), resident preference for SB (n=3), perceptions of clients/oneself being lazy/motivation (n=3).<br>- Skills: Lack of initiation and motor skill deficits (n=1).<br>- PA behaviour: Negative past experiences (n=3).<br>- Other behaviour: screen time (n=3).<br>-- INTERPERSONAL<br>- Staff: Staff's attitude (n=4), Staff's commitment (n=2), Staff as role models (n=1), low expectations of residents (n=3), staff lack of confidence in promotion (n=3), staff lack of interest (n=2), staff/client/family resistance (n=1).<br>-- ENVIRONMENTAL:<br>- Layout: Lack of space (n=2).		

				- Equipment: Lack of PA resources (n=2).<br>- Neighbourhood: Urban area (n=1), inaccessible neighborhoods (n=1), no sidewalks (n=1), too much traffic (n=1).<br>- Local amenities: Lack of nearby parks (n=1).<br>- Fitness amenities: Lack of tailored programs (n=3).<br>- Weather (n=3).<br>-- ORGANISATIONAL:<br>- Policy: PA as part of daily routine (n=2), lack of transportation (n=3), lack of clear policies (n=3), lack of same-sex staff members (n=1).<br>- Program offerings: Discontinued programs (n=4).<br>- Staff education: Lack of staff development program (n=2), lack of staff knowledge/creativity (n=4).<br>- Funding: Lack of funding (n=3).<br>- Culture: Lacking authority (n=3), staff past experiences promoting PA (n=2), PA promotion not a core part (n=1), inadequate staff-client ratios (n=8), prioritizing safety over PA (n=5), ageing staff (n=1), busy schedule/lack of time for PA (n=4), PA not prioritized (n=3), high staff turnover (n=2).		
**Author, publication year: MacDonald (2021)**<br>**Review aim: Review physical activity promotion interventions among individuals with intellectual disability and provide recommendations for increasing physical activity**						
**Review characteristics**<br>*(databases, search date)*	**Characteristics of included studies**<br>(number of studies, design, country, setting)	**Participant characteristics**	**Results**	**Influencing factors**	**Conclusion**	**Comments**
Systematic mapping review.	5 studies included.	Adults with intellectual disability,	Intrapersonal: Empowering is a key component to	Intrapersonal barriers:<br>- Job-related conflicts.	Studies used physical activity promotion	

Databases: MEDLINE, PsycINFO, SCOPUS.<br><br>Search date > July 2018.	Design: Quantitative (n=3), mixed methods (n=1), pre-post delayed (n=1).<br><br>Country: Not reported.<br><br>Setting: Not reported.	ranging from 19 to 65 years old.	facilitating health and physical activity promotion.<br><br>Interpersonal: Relationship are important sources of influence.<br><br>Organisational: Importance of structure and encouragement in schedules.	- Parental fears of participants getting sick.<br>- Multicomponent programs are complex.<br><br>Interpersonal facilitators:<br>- Involving family, friends and caregivers.<br>- Social support, also to arrange transportation etc.<br>- Praise and encouragement.<br><br>Interpersonal barriers:<br>- Finding time for participants and caregivers.<br>- Caregivers need more guidance, knowledge, "know how".<br><br>Organisational facilitators:<br>- Encouragement for physical activity in breaks.<br>- Structuring activities.	strategies at the intrapersonal, interpersonal, organisational, community, and policy levels have been used to date. Recommendations are presented for researchers and practitioners seeking to increase the level of PA of adults with intellectual disability.	
**Author, publication year: Maurer (2019)**<br>**Review aim: Identify the attitudes and needs of nursing home residents regarding physical activity**						
**Review characteristics**<br>*(databases, search date)*	**Characteristics of included studies**<br>(number of studies, design, country, setting)	**Participant characteristics**	**Results**	**Influencing factors**	**Conclusion**	**Comments**
Systematic thematic analysis.<br><br>Databases: MEDLINE/PubMed, PsycINFO, CINAHL.<br><br>Search date > January 2018.	12 studies included 404 residents.<br><br>Design: Qualitative (n=12).<br><br>Country: Germany (n=1), Taiwan (n=2), Norway (n=3), Ireland (n=1), Australia (n=2), Sweden (n=1), Austria (n=1), United Kingdom (n=1).	Nursing home residents, staff and significant others.	ATTITUDES<br>Personal initiative is significant:<br>- Seeing physical activity as own responsibility.<br>- Motivation: Set own goals and participate.<br>- A positive attitude towards living in a long-term care facility.<br>- Knowledge: Recognizing significance of physical	Barriers:<br>- Passive attitude/ resign to fate.<br>- Believe that physical activity does not help.<br>- Past negative experiences (muscle ache or fatigue).<br>- Afraid that it is harmful for their health.<br>- Disease-related physical symptoms.<br>- Mental illness.<br>- Not wanting to show weakness in front of other residents.<br>- Lack of information.	The identification of the main themes serve to better understand the attitudes (personal initiative is important; accepting the conditions; being physically active increases the quality of life;	

	Setting: Nursing homes (n=12).		activity to maintain abilities and skills.<br><br>Accepting the conditions:<br>- Positive: accepting need for support.<br>- Negative: Passive/ resign to fate.<br><br>Being physically active increases quality of life:<br>- Taking control of life.<br>- Realization that it helps to stay fit.<br><br>Being physically active is not helpful:<br>- Believe that physical activity does not help.<br>- Past negative experiences (muscle ache or fatigue).<br>- Afraid that it is harmful for their health.<br><br>NEEDS:<br>- Living autonomously.<br>- Continuing life as before.<br>- Competent care.<br>- Individually adapted program and support.<br>- Barrier-free accessibility.	- Accessibility: bad timing of classes or unsuitable location.<br><br>Facilitators:<br>- Seeing physical activity as own responsibility.<br>- Motivation: Set own goals and participate.<br>- A positive attitude towards living in a long-term care facility.<br>- Knowledge: Recognizing significance of physical activity to maintain abilities and skills.<br>- Positive: accepting need for support.<br>- Motivation to live independent and autonomously.<br>- Motivation to continue life (physical activity) as before.<br>- Extrinsic motivators, such as family members or a familiar group member.<br>- Activating aids, such as a transfer board or walker.<br>- Variety and adapted exercise programs.	being physically active is not helpful) and needs (living autonomously; continuing live as before; competent care; individually adapted program and support; barrier-free accessibility) of nursing home residents.	
**Author, publication year: Narsakka (2022)**<br>**Review aim: Synthesize evidence and provide a comprehensive understanding of the environmental aspects related to physical activity of older individuals in long-term care settings**						
**Review characteristics**	**Characteristics of included studies**	**Participant characteristics**	**Results**	**Influencing factors**	**Conclusion**	**Comments**

(databases, search date)	(number of studies, design, country, setting)					
Mixed-method systematic review.<br><br>Databases: CINAHL, PubMed, Cochrane PsycINFO<br><br>Search date > Dec 31 2020.	30 studies included.<br><br>Design: Qualitative studies (n=24) and mixed studies (n=9).<br><br>Country: Australia (n=1), Belgium (n=2), Canada (n=4), Finland (n=1), Germany (n=1), Norway (n=1), the Netherlands (n=3), Slovenia (n=1), Sweden (n=2), Taiwan (n=2), the UK (n=3), and the USA (n = 8).<br><br>Setting: nursing homes (n=10), residential care facilities (n=4), assisted living facilities (n=5), care homes (n=2), dementia care units (n=1), elderly care units (n=1), psychogeriatric nursing homes (n=1), and long-term care facilities (n=4).	Residents in long-term care facilities.	Physical environment:<br>- accessible and safe living environment.<br>- activating physical environment.<br><br>Social environment:<br>- supportive professionals.<br>- the role of other people.<br>- adequate activities to socialize and be active.<br><br>Symbolic environment:<br>- policy at multiple levels.<br>- values of organisations and professionals.	FACILITATORS:<br>Physical environment:<br>- Space to move, turn and pass.<br>- Wide doorways, automatic doors, functioning elevators.<br>- Well-lit areas, good lighting, brightness.<br>- Places to sit and rest.<br>- Shade.<br>- Safety: Handrails, mobility aids, proper signage to navigate, visual access for nurses to monitor residents, monitoring devices.<br>- Building environment: integration in a regular street, safe walkways outside, proximity to public facilities and gardens, outdoor areas (greenhouses and balconies).<br>- Independent access outdoors.<br>- Walking paths outdoors and indoors / floor plans / circular designs.<br>- Different wall colors, furniture, and carpets.<br>- Rooms dedicated to exercise.<br>- Interactive art work.<br><br>Social environment:<br>- Facilitating more independence in care duties.<br>- Opinion of professionals and residents' trust in them.<br>- Encouragement and social support.<br>- Being in contact with community.<br>- Family involvement and encouragement.<br>- Family members contributions and educating them.<br>- Volunteers that helped in supporting.<br>- Satisfaction with available activities.<br><br>Symbolic environment:<br>- Valuing maintaining of residents'	Facilitators and barriers were found for three theme's: physical environment, social environment, symbolic environment. Different environmental aspects within and between the dimensions of the physical, social, and symbolic environment were related to each other.	

				functioning.<br>- Support from facility's board and managerial process.<br>- More experience of staff.<br><br>BARRIERS:<br>Physical environment:<br>- Limited space to move.<br>- Heavy or difficult doors.<br>- Doorsteps, stairs, steep pathways, ramps.<br>- Characteristics of floor: shininess and color differences.<br>- Building environment: Busy roads next to the facilities.<br>- Dead end hallways and short corridors.<br>- Lack of space for exercise in group and self-initiated.<br>- Exercise rooms that are also used for other purpose.<br><br>Social environment:<br>- Strict schedules and mechanistic approaches.<br>- Lack of resources, such as lack of staff and financial resources.<br>- Care culture of facilities.<br>- Motivation of staff.<br>- Isolation of facilities from community.<br>- Family members worried.<br>- Lack of activities + Activities did not meet preferences or needs.<br><br>Symbolic environment:<br>- Laws and regulations for safety and hygiene.<br>- Locked door policies.<br>- Using equipment only under supervision.		

				- Unmotivated staff; Physical activity not valued.		
**Author, publication year: Vseteckova (2018)**<br>**Review aim: Collect and synthesize evidence on the known barriers and facilitators to adherence to group exercise of institutionalized older people living with dementia**						
**Review characteristics**<br>*(databases, search date)*	**Characteristics of included studies**<br>(number of studies, design, country, setting)	**Participant characteristics**	**Results**	**Influencing factors**	**Conclusion**	**Comments**
Systematic review.<br><br>Databases: MEDLINE, Cochrane, PsycINFO, ERIC, CINAHL, Web of Science, trial registers, SCOPUS, Google Scholar.<br><br>Search date: 1990 – Sept 2017.	9 studies included 1630 participants.<br><br>Design: Qualitative (n=3), randomized controlled trial (n=5), study protocol (n=1).<br><br>Country: Not reported.<br><br>Setting: Not reported.	Institutionalized older people living with dementia, worldwide.	BARRIERS<br>- Bio-medical reasons, mental wellbeing and physical ability.<br>- Relationships dynamics.<br>- Socioeconomic reasons.<br><br>FACILITATORS<br>- Bio-medical benefits and benefits related to physical ability.<br>- Feelings and emotions and confidence improvements.<br>- Therapist and group relationship dynamics.<br>- Activity related reasons.<br><br>Adherence ranged from 84% to 25.5% with high interindividual variability.	BARRIERS<br>Bio-medical reasons, mental wellbeing and physical ability:<br>- Acute disease (n=1).<br>- Anxiety and agitation, depression (n=2).<br>- Being cognitively more intact (n=1).<br>- Fear of injury (n=1).<br>- Frailty including symptoms of muscle weakness (n=1).<br>- Increased disability in ADL (n=1).<br>- Low levels of previous physical activity and slow walking speed (n=1).<br>- Medication (n=1).<br><br>Relationships dynamics<br>- Disagreement within the groups or unwillingness to continue (n=1).<br>- Family expectations and communications (n=1).<br><br>Socioeconomic reasons<br>- Low staffing levels (n=2).<br>- Socioeconomic status, the lower the more of a barrier (n=1).<br><br>FACILITATORS<br>Bio-medical benefits and benefits related to physical ability<br>- Physiological benefits, improvements in physical well-being, pushing the limits	We conclude that institutionalized older people living with dementia, even those who are physically frail, incontinent and/or have mild dementia can demonstrate certain level of exercise adherence, and therefore can respond positively to exercise programs. Tailored, individually-adjusted and supported physical activity, led by a knowledgeable, engaging and well communicating therapist/facilitator improves the adherence to group exercise interventions of institutionalized	

				(n=2).<br>- Skills improvement (n=2).<br><br>Feelings and emotions and confidence improvements<br>- Mastery of exercise, empowerment, psychological well-being, self-worth, enjoyment and achievement linked to self-efficacy (n=2).<br>- Regaining control and increased independence and improved self-esteem (n=2).<br><br>Therapist and group relationship dynamics<br>- Anticipating challenges, being prepared; giving written instruction where necessary; using assistive devices where necessary (n=2).<br>- Availability of staff (n=1).<br>- Knowing the persons's past; humour and play; short clear verbal cues; repetition; communicating; giving attention (n=2).<br>- Motivating nursing assistants as well as the residents to engage with physical activities (n=2).<br>- Presence of an activity coordinator or therapist and their competence, trustworthiness and knowledgeability (n=2).<br><br>Activity related reasons<br>- Allowing space for gaming approach (light competition for example) where appropriate in a socially encouraging environment (n=1).<br>- Flexible scheduling and voluntary participation (n=3).<br>- Tailoring the activity and its safeness (n=3), setting realistic individual goals and targeting improving independence (n=1),	older people living with dementia.	

				moderating appropriate activity dosage whenever appropriate (n=2), allowing space for individuals uptake of the exercise (n=1), challenging if necessary (n=1).		

Risk of bias tables

AMSTAR-2 assessments per study on physical activities (n=6; module 'Physical activities').

Author, publication year: Barrett 2020	Judgement	Low quality review
Item	Yes, partial yes or no	Explanation
Did the research questions and inclusion criteria for the review include the components of PICO?	Yes	
Did the report of the review contain an explicit statement that the review methods were established prior to the conduct of the review and did the report justify any significant deviations from the protocol?	Yes	
Did the review authors explain their selection of the study designs for inclusion in the review?	Yes	
Did the review authors use a comprehensive literature search strategy?	Yes	
Did the review authors perform study selection in duplicate?	No	Second reviewer was included in case of uncertainty
Did the review authors perform data extraction in duplicate?	Yes	
Did the review authors provide a list of excluded studies and justify the exclusions?	No	Not reported, reasons reported in the PRISMA flowchart
Did the review authors describe the included studies in adequate detail?	Yes	
Did the review authors use a satisfactory technique for assessing the risk of bias (RoB) in individual studies that were included in the review?	Yes	
Did the review authors report on the sources of funding for the studies included in the review?	Yes	
If meta-analysis was performed did the review authors use appropriate methods for statistical combination of results?	N/A	No meta-analysis
If meta-analysis was performed, did the review authors assess the potential impact of RoB in individual studies on the results of the meta-analysis or other evidence synthesis?	N/A	No meta-analysis
Did the review authors account for RoB in individual studies when interpreting/ discussing the results of the review?	Yes	
Did the review authors provide a satisfactory explanation for, and discussion of, any heterogeneity observed in the results of the review?	No	Not discussed in the discussion section
If they performed quantitative synthesis did the review authors carry out an adequate investigation of publication bias (small study bias) and discuss its likely impact on the results of the review?	N/A	No meta-analysis
Did the review authors report any potential sources of conflict of interest, including any funding they received for conducting the review?	Yes	

Author, publication year: Brett et al 2016	Judgement	Critically low quality
Item	Yes, partial yes or no	Explanation
Did the research questions and inclusion criteria for the review include the components of PICO?	Yes	
Did the report of the review contain an explicit statement that the review methods were established prior to the conduct of the review and did the report justify any significant deviations from the protocol?	No	No protocol published.
Did the review authors explain their selection of the study designs for inclusion in the review?	Yes	
Did the review authors use a comprehensive literature search strategy?	Yes	

Did the review authors perform study selection in duplicate?	No	Only 1 extractor, but checked by second and third author.
Did the review authors perform data extraction in duplicate?	No	Only 1 extractor, but checked by second and third author.
Did the review authors provide a list of excluded studies and justify the exclusions?	No	Not reported.
Did the review authors describe the included studies in adequate detail?	Yes	
Did the review authors use a satisfactory technique for assessing the risk of bias (RoB) in individual studies that were included in the review?	Yes	
Did the review authors report on the sources of funding for the studies included in the review?	Yes	
If meta-analysis was performed did the review authors use appropriate methods for statistical combination of results?	N/A	No meta-analysis
If meta-analysis was performed, did the review authors assess the potential impact of RoB in individual studies on the results of the meta-analysis or other evidence synthesis?	N/A	No meta-analysis
Did the review authors account for RoB in individual studies when interpreting/ discussing the results of the review?	Yes	
Did the review authors provide a satisfactory explanation for, and discussion of, any heterogeneity observed in the results of the review?	No	Not discussed in the discussion section
If they performed quantitative synthesis did the review authors carry out an adequate investigation of publication bias (small study bias) and discuss its likely impact on the results of the review?	N/A	No meta-analysis
Did the review authors report any potential sources of conflict of interest, including any funding they received for conducting the review?	Yes	

Author, publication year: Brooker et al 2015	Judgement	Critically low quality
Item	Yes, partial yes or no	Explanation
Did the research questions and inclusion criteria for the review include the components of PICO?	Yes	
Did the report of the review contain an explicit statement that the review methods were established prior to the conduct of the review and did the report justify any significant deviations from the protocol?	No	No protocol.
Did the review authors explain their selection of the study designs for inclusion in the review?	Yes	
Did the review authors use a comprehensive literature search strategy?	Yes	
Did the review authors perform study selection in duplicate?	Yes	
Did the review authors perform data extraction in duplicate?	Yes	
Did the review authors provide a list of excluded studies and justify the exclusions?	No	Not reported.
Did the review authors describe the included studies in adequate detail?	Yes	
Did the review authors use a satisfactory technique for assessing the risk of bias (RoB) in individual studies that were included in the review?	No	They did not use a validated tool.
Did the review authors report on the sources of funding for the studies included in the review?	No	Not reported.
If meta-analysis was performed did the review authors use appropriate methods for statistical combination of results?	N/A	No meta-analysis
If meta-analysis was performed, did the review authors assess the potential impact of RoB in individual studies on the results of the meta-analysis or other evidence synthesis?	N/A	No meta-analysis
Did the review authors account for RoB in individual studies when interpreting/ discussing the results of the review?	No	Not reported

Did the review authors provide a satisfactory explanation for, and discussion of, any heterogeneity observed in the results of the review?	No	Not discussed in the discussion section
If they performed quantitative synthesis did the review authors carry out an adequate investigation of publication bias (small study bias) and discuss its likely impact on the results of the review?	N/A	No meta-analysis
Did the review authors report any potential sources of conflict of interest, including any funding they received for conducting the review?	No	Not reported.

Author, publication year: Jansen et al 2015	Judgement	Critically low quality
Item	Yes, partial yes or no	Explanation
Did the research questions and inclusion criteria for the review include the components of PICO?	Yes	
Did the report of the review contain an explicit statement that the review methods were established prior to the conduct of the review and did the report justify any significant deviations from the protocol?	No	No protocol published.
Did the review authors explain their selection of the study designs for inclusion in the review?	No	Not reported why they only included RCTs
Did the review authors use a comprehensive literature search strategy?	Yes	
Did the review authors perform study selection in duplicate?	Yes	
Did the review authors perform data extraction in duplicate?	Yes	
Did the review authors provide a list of excluded studies and justify the exclusions?	No	Only gave summarized reasons but not per study.
Did the review authors describe the included studies in adequate detail?	Yes	
Did the review authors use a satisfactory technique for assessing the risk of bias (RoB) in individual studies that were included in the review?	Yes	
Did the review authors report on the sources of funding for the studies included in the review?	No	Not reported
If meta-analysis was performed did the review authors use appropriate methods for statistical combination of results?	N/A	No meta-analysis
If meta-analysis was performed, did the review authors assess the potential impact of RoB in individual studies on the results of the meta-analysis or other evidence synthesis?	N/A	No meta-analysis
Did the review authors account for RoB in individual studies when interpreting/ discussing the results of the review?	No	Not reported.
Did the review authors provide a satisfactory explanation for, and discussion of, any heterogeneity observed in the results of the review?	No	Not discussed in the discussion section
If they performed quantitative synthesis did the review authors carry out an adequate investigation of publication bias (small study bias) and discuss its likely impact on the results of the review?	N/A	No meta-analysis
Did the review authors report any potential sources of conflict of interest, including any funding they received for conducting the review?	No	Not reported

Author, publication year: Temple et al 2017	Judgement	Critically low quality
Item	Yes, partial yes or no	Explanation
Did the research questions and inclusion criteria for the review include the components of PICO?	Yes	
Did the report of the review contain an explicit statement that the review methods were established prior to the conduct of the review and did the report justify any significant deviations from the protocol?	No	No protocol published.

Did the review authors explain their selection of the study designs for inclusion in the review?	Yes	
Did the review authors use a comprehensive literature search strategy?	Yes	
Did the review authors perform study selection in duplicate?	No	Not reported.
Did the review authors perform data extraction in duplicate?	No	Not reported.
Did the review authors provide a list of excluded studies and justify the exclusions?	No	Not reported.
Did the review authors describe the included studies in adequate detail?	Yes	
Did the review authors use a satisfactory technique for assessing the risk of bias (RoB) in individual studies that were included in the review?	No	No RoB was conducted.
Did the review authors report on the sources of funding for the studies included in the review?	No	Not reported.
If meta-analysis was performed did the review authors use appropriate methods for statistical combination of results?	N/A	No meta-analysis
If meta-analysis was performed, did the review authors assess the potential impact of RoB in individual studies on the results of the meta-analysis or other evidence synthesis?	N/A	No meta-analysis
Did the review authors account for RoB in individual studies when interpreting/ discussing the results of the review?	No	No RoB.
Did the review authors provide a satisfactory explanation for, and discussion of, any heterogeneity observed in the results of the review?	No	Not discussed in the discussion section
If they performed quantitative synthesis did the review authors carry out an adequate investigation of publication bias (small study bias) and discuss its likely impact on the results of the review?	N/A	No meta-analysis
Did the review authors report any potential sources of conflict of interest, including any funding they received for conducting the review?	Yes	

Author, publication year: Wylie et al 2023	Judgement	Critically low
Item	Yes, partial yes or no	Explanation
Did the research questions and inclusion criteria for the review include the components of PICO?	Yes	
Did the report of the review contain an explicit statement that the review methods were established prior to the conduct of the review and did the report justify any significant deviations from the protocol?	No	Not reported.
Did the review authors explain their selection of the study designs for inclusion in the review?	Yes	
Did the review authors use a comprehensive literature search strategy?	Yes	
Did the review authors perform study selection in duplicate?	No	Each author screened a phase independently.
Did the review authors perform data extraction in duplicate?	No	Not reported.
Did the review authors provide a list of excluded studies and justify the exclusions?	No	Not reported.
Did the review authors describe the included studies in adequate detail?	Yes	
Did the review authors use a satisfactory technique for assessing the risk of bias (RoB) in individual studies that were included in the review?	Yes	
Did the review authors report on the sources of funding for the studies included in the review?	Yes	
If meta-analysis was performed did the review authors use appropriate methods for statistical combination of results?	N/A	No meta-analysis

If meta-analysis was performed, did the review authors assess the potential impact of RoB in individual studies on the results of the meta-analysis or other evidence synthesis?	N/A	No meta-analysis
Did the review authors account for RoB in individual studies when interpreting/ discussing the results of the review?	No	Not reported.
Did the review authors provide a satisfactory explanation for, and discussion of, any heterogeneity observed in the results of the review?	N/A	No meta-analysis
If they performed quantitative synthesis did the review authors carry out an adequate investigation of publication bias (small study bias) and discuss its likely impact on the results of the review?	N/A	No meta-analysis
Did the review authors report any potential sources of conflict of interest, including any funding they received for conducting the review?	Yes	

AMSTAR-2 assessments per study on technology (n=8; module 'Technology').

**Author, publication year: Agbangla, 2022**		
**Item**	**Yes, partial yes or no**	**Explanation**
1. Did the research questions and inclusion criteria for the review include the components of PICO?	Yes	Population and interventions clearly described. Outcomes and comparators not clear in the methods, but these are described in the results.
2. Did the report of the review contain an explicit statement that the review methods were established prior to the conduct of the review and did the report justify any significant deviations from the protocol?	No	
3. Did the review authors explain their selection of the study designs for inclusion in the review?	No	
4. Did the review authors use a comprehensive literature search strategy?	Partial	
5. Did the review authors perform study selection in duplicate?	Yes	
6. Did the review authors perform data extraction in duplicate?	No	Second researcher checked but kappa score not reported
7. Did the review authors provide a list of excluded studies and justify the exclusions?	No	
8. Did the review authors describe the included studies in adequate detail?	Partial yes	No details
9. Did the review authors use a satisfactory technique for assessing the risk of bias in individual studies that were included in the review?	No	
10. Did the review authors report on the sources of funding for the studies included in the review?	No	
11. If meta-analyses was performed did the review authors use appropriate methods for statistical combination of results?	NA	
12. If meta-analysis was performed, did the review authors assess the potential impact of risk of bias in individual studies on the results of the meta-analysis or other evidence synthesis?	NA	
13. Did the review authors account for risk of bias in individual studies when interpreting/discussing the results of the review?	No	
14. Did the review authors provide a satisfactory explanation for, and discussion of, any heterogeneity observed in the results of the review?	No	Limited discussion
15. If they performed quantitative synthesis did the review authors carry out an adequate investigation of publication bias (small study bias) and discuss its likely impact on the results of the review?	No	
16. Did the review authors report any potential sources of conflict of interest, including any funding they received for conducting the review?	Yes	

**Author, publication year: Chen, 2023**		

Item	Yes, partial yes or no	Explanation
1. Did the research questions and inclusion criteria for the review include the components of PICO?	Yes	
2. Did the report of the review contain an explicit statement that the review methods were established prior to the conduct of the review and did the report justify any significant deviations from the protocol?	Yes	Registered as PROSPERO (CRD42021241923)
3. Did the review authors explain their selection of the study designs for inclusion in the review?	No	RCTs included but no explanation
4. Did the review authors use a comprehensive literature search strategy?	Partial yes	Reference lists were searched. No grey literature etc
5. Did the review authors perform study selection in duplicate?	Yes	
6. Did the review authors perform data extraction in duplicate?	No	One researcher extracted data, the others checked. Kappa score not reported.
7. Did the review authors provide a list of excluded studies and justify the exclusions?	No	
8. Did the review authors describe the included studies in adequate detail?	Partial	Comparator and study settings not in detail
9. Did the review authors use a satisfactory technique for assessing the risk of bias in individual studies that were included in the review?	Yes	Cochrane tool + GRADE
10. Did the review authors report on the sources of funding for the studies included in the review?	No	Not reported per study
11. If meta-analyses was performed did the review authors use appropriate methods for statistical combination of results?	Yes	
12. If meta-analysis was performed, did the review authors assess the potential impact of risk of bias in individual studies on the results of the meta-analysis or other evidence synthesis?	No	One-by-one sensitivity analysis was conducted, but no subgroup analysis for high-risk studies
13. Did the review authors account for risk of bias in individual studies when interpreting/discussing the results of the review?	No	
14. Did the review authors provide a satisfactory explanation for, and discussion of, any heterogeneity observed in the results of the review?	No	Heterogeneity was measured but not explained
15. If they performed quantitative synthesis did the review authors carry out an adequate investigation of publication bias (small study bias) and discuss its likely impact on the results of the review?	Yes	
16. Did the review authors report any potential sources of conflict of interest, including any funding they received for conducting the review?	Yes	

Author, publication year: Chu, 2022		
**Item**	**Yes, partial yes or no**	**Explanation**
1. Did the research questions and inclusion criteria for the review include the components of PICO?	Yes	
2. Did the report of the review contain an explicit statement that the review methods were established prior to the conduct of the review and did the report justify any significant deviations from the protocol?	No	
3. Did the review authors explain their selection of the study designs for inclusion in the review?	No	
4. Did the review authors use a comprehensive literature search strategy?	Partial	References lists were searched. Not all grey literature
5. Did the review authors perform study selection in duplicate?	Yes	
6. Did the review authors perform data extraction in duplicate?	No	
7. Did the review authors provide a list of excluded studies and justify the exclusions?	No	
8. Did the review authors describe the included studies in adequate detail?	Yes	
9. Did the review authors use a satisfactory technique for assessing the risk of bias in individual studies that were included in the review?	Yes	Cochrane Rob-2 + ROBINS

10. Did the review authors report on the sources of funding for the studies included in the review?	No	
11. If meta-analyses was performed did the review authors use appropriate methods for statistical combination of results?	NA	
12. If meta-analysis was performed, did the review authors assess the potential impact of risk of bias in individual studies on the results of the meta-analysis or other evidence synthesis?	NA	
13. Did the review authors account for risk of bias in individual studies when interpreting/discussing the results of the review?	No	
14. Did the review authors provide a satisfactory explanation for, and discussion of, any heterogeneity observed in the results of the review?	No	
15. If they performed quantitative synthesis did the review authors carry out an adequate investigation of publication bias (small study bias) and discuss its likely impact on the results of the review?	No	
16. Did the review authors report any potential sources of conflict of interest, including any funding they received for conducting the review?	Yes	

**Author, publication year: Diener, 2022**		
**Item**	**Yes, partial yes or no**	**Explanation**
1. Did the research questions and inclusion criteria for the review include the components of PICO?	Yes	
2. Did the report of the review contain an explicit statement that the review methods were established prior to the conduct of the review and did the report justify any significant deviations from the protocol?	Yes	PROSPERO: CRD42021289488
3. Did the review authors explain their selection of the study designs for inclusion in the review?	No	
4. Did the review authors use a comprehensive literature search strategy?	Partial	Reference lists etc were searched. No experts / further grey literature
5. Did the review authors perform study selection in duplicate?	Yes	
6. Did the review authors perform data extraction in duplicate?	No	Second researcher verified, no kappa score reported.
7. Did the review authors provide a list of excluded studies and justify the exclusions?	No	
8. Did the review authors describe the included studies in adequate detail?	Partial	Not in detail
9. Did the review authors use a satisfactory technique for assessing the risk of bias in individual studies that were included in the review?	Partial	
10. Did the review authors report on the sources of funding for the studies included in the review?	No	
11. If meta-analyses was performed did the review authors use appropriate methods for statistical combination of results?	NA	
12. If meta-analysis was performed, did the review authors assess the potential impact of risk of bias in individual studies on the results of the meta-analysis or other evidence synthesis?	NA	
13. Did the review authors account for risk of bias in individual studies when interpreting/discussing the results of the review?	No	
14. Did the review authors provide a satisfactory explanation for, and discussion of, any heterogeneity observed in the results of the review?	No	
15. If they performed quantitative synthesis did the review authors carry out an adequate investigation of publication bias (small study bias) and discuss its likely impact on the results of the review?	No	
16. Did the review authors report any potential sources of conflict of interest, including any funding they received for conducting the review?	Yes	

**Author, publication year: Kukkohovi, 2023**		

Item	Yes, partial yes or no	Explanation
1. Did the research questions and inclusion criteria for the review include the components of PICO?	Yes	
2. Did the report of the review contain an explicit statement that the review methods were established prior to the conduct of the review and did the report justify any significant deviations from the protocol?	Partial	PROSPERO: CRD42022307491. No plan for hetereogeneity
3. Did the review authors explain their selection of the study designs for inclusion in the review?	No	
4. Did the review authors use a comprehensive literature search strategy?	Partial	Manual search without further details
5. Did the review authors perform study selection in duplicate?	Yes	
6. Did the review authors perform data extraction in duplicate?	No	Second researcher checked. No kappa score reported.
7. Did the review authors provide a list of excluded studies and justify the exclusions?	No	
8. Did the review authors describe the included studies in adequate detail?	Yes	Described in detail, setting less clear
9. Did the review authors use a satisfactory technique for assessing the risk of bias in individual studies that were included in the review?	Yes	Joanna Briggs Institute
10. Did the review authors report on the sources of funding for the studies included in the review?	No	
11. If meta-analyses was performed did the review authors use appropriate methods for statistical combination of results?	Yes	
12. If meta-analysis was performed, did the review authors assess the potential impact of risk of bias in individual studies on the results of the meta-analysis or other evidence synthesis?	No	
13. Did the review authors account for risk of bias in individual studies when interpreting/discussing the results of the review?	No	
14. Did the review authors provide a satisfactory explanation for, and discussion of, any heterogeneity observed in the results of the review?	No	
15. If they performed quantitative synthesis did the review authors carry out an adequate investigation of publication bias (small study bias) and discuss its likely impact on the results of the review?	No	Could not be assessed due to small number of studies per outcome
16. Did the review authors report any potential sources of conflict of interest, including any funding they received for conducting the review?	Yes	

Author, publication year: Lancioni, 2022		
**Item**	**Yes, partial yes or no**	**Explanation**
1. Did the research questions and inclusion criteria for the review include the components of PICO?	Yes	
2. Did the report of the review contain an explicit statement that the review methods were established prior to the conduct of the review and did the report justify any significant deviations from the protocol?	No	
3. Did the review authors explain their selection of the study designs for inclusion in the review?	No	
4. Did the review authors use a comprehensive literature search strategy?	Partial yes	Google scholar and reference lists were searched
5. Did the review authors perform study selection in duplicate?	No	
6. Did the review authors perform data extraction in duplicate?	Yes	
7. Did the review authors provide a list of excluded studies and justify the exclusions?	No	
8. Did the review authors describe the included studies in adequate detail?	Yes	Multimedia appendix 1 studies in detail.
9. Did the review authors use a satisfactory technique for assessing the risk of bias in individual studies that were included in the review?	No	
10. Did the review authors report on the sources of funding for the studies included in the review?	No	

11. If meta-analyses was performed did the review authors use appropriate methods for statistical combination of results?	NA	
12. If meta-analysis was performed, did the review authors assess the potential impact of risk of bias in individual studies on the results of the meta-analysis or other evidence synthesis?	NA	
13. Did the review authors account for risk of bias in individual studies when interpreting/discussing the results of the review?	No	
14. Did the review authors provide a satisfactory explanation for, and discussion of, any heterogeneity observed in the results of the review?	No	
15. If they performed quantitative synthesis did the review authors carry out an adequate investigation of publication bias (small study bias) and discuss its likely impact on the results of the review?	NA	
16. Did the review authors report any potential sources of conflict of interest, including any funding they received for conducting the review?	No	

Author, publication year: Li, 2023		
**Item**	**Yes, partial yes or no**	**Explanation**
1. Did the research questions and inclusion criteria for the review include the components of PICO?	Yes	
2. Did the report of the review contain an explicit statement that the review methods were established prior to the conduct of the review and did the report justify any significant deviations from the protocol?	Partial	PROSPERO: CRD42022323533
3. Did the review authors explain their selection of the study designs for inclusion in the review?	No	
4. Did the review authors use a comprehensive literature search strategy?	Partial	Reference lists of included studies were searched
5. Did the review authors perform study selection in duplicate?	Yes	
6. Did the review authors perform data extraction in duplicate?	Yes	
7. Did the review authors provide a list of excluded studies and justify the exclusions?	No	
8. Did the review authors describe the included studies in adequate detail?	Partial	Comparator and setting not in detail
9. Did the review authors use a satisfactory technique for assessing the risk of bias in individual studies that were included in the review?	Yes	Cochrane Risk of Bias tool
10. Did the review authors report on the sources of funding for the studies included in the review?	No	
11. If meta-analyses was performed did the review authors use appropriate methods for statistical combination of results?	NA	
12. If meta-analysis was performed, did the review authors assess the potential impact of risk of bias in individual studies on the results of the meta-analysis or other evidence synthesis?	NA	
13. Did the review authors account for risk of bias in individual studies when interpreting/discussing the results of the review?	No	
14. Did the review authors provide a satisfactory explanation for, and discussion of, any heterogeneity observed in the results of the review?	No	No discussion, it was mentioned in limitations
15. If they performed quantitative synthesis did the review authors carry out an adequate investigation of publication bias (small study bias) and discuss its likely impact on the results of the review?	No	
16. Did the review authors report any potential sources of conflict of interest, including any funding they received for conducting the review?	Yes	

Author, publication year: Swinnen, 2022		
**Item**	**Yes, partial yes or no**	**Explanation**
1. Did the research questions and inclusion criteria for the review include the components of PICO?	Yes	

2. Did the report of the review contain an explicit statement that the review methods were established prior to the conduct of the review and did the report justify any significant deviations from the protocol?	Partial	PROSPERO: CRD42020156737
3. Did the review authors explain their selection of the study designs for inclusion in the review?	Yes	In the PROSPERO protocol
4. Did the review authors use a comprehensive literature search strategy?	Partial	Manual searches. Not all options conducted
5. Did the review authors perform study selection in duplicate?	Yes	
6. Did the review authors perform data extraction in duplicate?	Yes	
7. Did the review authors provide a list of excluded studies and justify the exclusions?	Yes	
8. Did the review authors describe the included studies in adequate detail?	Yes	
9. Did the review authors use a satisfactory technique for assessing the risk of bias in individual studies that were included in the review?	Yes	ROBINS-I en GRADE
10. Did the review authors report on the sources of funding for the studies included in the review?	No	
11. If meta-analyses was performed did the review authors use appropriate methods for statistical combination of results?	NA	
12. If meta-analysis was performed, did the review authors assess the potential impact of risk of bias in individual studies on the results of the meta-analysis or other evidence synthesis?	NA	
13. Did the review authors account for risk of bias in individual studies when interpreting/discussing the results of the review?	No	
14. Did the review authors provide a satisfactory explanation for, and discussion of, any heterogeneity observed in the results of the review?	No	
15. If they performed quantitative synthesis did the review authors carry out an adequate investigation of publication bias (small study bias) and discuss its likely impact on the results of the review?	No	
16. Did the review authors report any potential sources of conflict of interest, including any funding they received for conducting the review?	No	

AMSTAR-2 assessments per study on motivation (n=11, modules 'Motivating clients', 'Encouraging informal caregivers' and 'Organisation of care').

**Author, publication year: Aitchison, 2022**		
**Item**	**Yes, partial yes or no**	**Explanation**
1. Did the research questions and inclusion criteria for the review include the components of PICO?	Yes	
2. Did the report of the review contain an explicit statement that the review methods were established prior to the conduct of the review and did the report justify any significant deviations from the protocol?	Yes	No plan for heterogeneity, but yes because it is a qualitative analysis
3. Did the review authors explain their selection of the study designs for inclusion in the review?	No	All studies included, no explanation
4. Did the review authors use a comprehensive literature search strategy?	Partial yes	Comprehensive search with grey literature.
5. Did the review authors perform study selection in duplicate?	Yes	
6. Did the review authors perform data extraction in duplicate?	No	
7. Did the review authors provide a list of excluded studies and justify the exclusions?	No	
8. Did the review authors describe the included studies in adequate detail?	Partial yes	
9. Did the review authors use a satisfactory technique for assessing the risk of bias in individual studies that were included in the review?	Yes	QATSDD + GRADECERQual were used
10. Did the review authors report on the sources of funding for the studies included in the review?	No	
11. If meta-analyses was performed did the review authors use appropriate methods for statistical combination of results?	NA	

12. If meta-analysis was performed, did the review authors assess the potential impact of risk of bias in individual studies on the results of the meta-analysis or other evidence synthesis?	NA	
13. Did the review authors account for risk of bias in individual studies when interpreting/discussing the results of the review?	No	GRADE-CERQual was used so it was taken into account in a way. Not explicitly.
14. Did the review authors provide a satisfactory explanation for, and discussion of, any heterogeneity observed in the results of the review?	No	
15. If they performed quantitative synthesis did the review authors carry out an adequate investigation of publication bias (small study bias) and discuss its likely impact on the results of the review?	NA	
16. Did the review authors report any potential sources of conflict of interest, including any funding they received for conducting the review?	No	

**Author, publication year: Anderiesen, 2014**		
**Item**	**Yes, partial yes or no**	**Explanation**
1. Did the research questions and inclusion criteria for the review include the components of PICO?	Yes	
2. Did the report of the review contain an explicit statement that the review methods were established prior to the conduct of the review and did the report justify any significant deviations from the protocol?	No	
3. Did the review authors explain their selection of the study designs for inclusion in the review?	No	
4. Did the review authors use a comprehensive literature search strategy?	Partial yes	No grey literature
5. Did the review authors perform study selection in duplicate?	Yes	First author reviewed all, and two researcher did duplicate
6. Did the review authors perform data extraction in duplicate?	No	
7. Did the review authors provide a list of excluded studies and justify the exclusions?	No	
8. Did the review authors describe the included studies in adequate detail?	Partial yes	Focus on results. Individual studies not adequately described.
9. Did the review authors use a satisfactory technique for assessing the risk of bias in individual studies that were included in the review?	Partial yes	
10. Did the review authors report on the sources of funding for the studies included in the review?	No	
11. If meta-analyses was performed did the review authors use appropriate methods for statistical combination of results?	NA	
12. If meta-analysis was performed, did the review authors assess the potential impact of risk of bias in individual studies on the results of the meta-analysis or other evidence synthesis?	NA	
13. Did the review authors account for risk of bias in individual studies when interpreting/discussing the results of the review?	Yes	
14. Did the review authors provide a satisfactory explanation for, and discussion of, any heterogeneity observed in the results of the review?	No	
15. If they performed quantitative synthesis did the review authors carry out an adequate investigation of publication bias (small study bias) and discuss its likely impact on the results of the review?	NA	
16. Did the review authors report any potential sources of conflict of interest, including any funding they received for conducting the review?	No	

**Author, publication year: Benjamin, 2014**		
**Item**	**Yes, partial yes or no**	**Explanation**
1. Did the research questions and inclusion criteria for the review include the components of PICO?	Yes	

2. Did the report of the review contain an explicit statement that the review methods were established prior to the conduct of the review and did the report justify any significant deviations from the protocol?	No	
3. Did the review authors explain their selection of the study designs for inclusion in the review?	Yes	
4. Did the review authors use a comprehensive literature search strategy?	Partial yes	
5. Did the review authors perform study selection in duplicate?	No	
6. Did the review authors perform data extraction in duplicate?	No	
7. Did the review authors provide a list of excluded studies and justify the exclusions?	No	
8. Did the review authors describe the included studies in adequate detail?	No	
9. Did the review authors use a satisfactory technique for assessing the risk of bias in individual studies that were included in the review?	No	Questions from a tool of Polit and Beck were used
10. Did the review authors report on the sources of funding for the studies included in the review?	No	
11. If meta-analyses was performed did the review authors use appropriate methods for statistical combination of results?	NA	
12. If meta-analysis was performed, did the review authors assess the potential impact of risk of bias in individual studies on the results of the meta-analysis or other evidence synthesis?	NA	
13. Did the review authors account for risk of bias in individual studies when interpreting/discussing the results of the review?	No	
14. Did the review authors provide a satisfactory explanation for, and discussion of, any heterogeneity observed in the results of the review?	No	
15. If they performed quantitative synthesis did the review authors carry out an adequate investigation of publication bias (small study bias) and discuss its likely impact on the results of the review?	NA	
16. Did the review authors report any potential sources of conflict of interest, including any funding they received for conducting the review?	No	

**Author, publication year: Bossink, 2017**		
**Item**	**Yes, partial yes or no**	**Explanation**
1. Did the research questions and inclusion criteria for the review include the components of PICO?	Yes	
2. Did the report of the review contain an explicit statement that the review methods were established prior to the conduct of the review and did the report justify any significant deviations from the protocol?	No	
3. Did the review authors explain their selection of the study designs for inclusion in the review?	No	
4. Did the review authors use a comprehensive literature search strategy?	Partial yes	References lists and "cited by" were checked.
5. Did the review authors perform study selection in duplicate?	Yes	Agreement was checked
6. Did the review authors perform data extraction in duplicate?	No	
7. Did the review authors provide a list of excluded studies and justify the exclusions?	No	
8. Did the review authors describe the included studies in adequate detail?	Yes	
9. Did the review authors use a satisfactory technique for assessing the risk of bias in individual studies that were included in the review?	Partial yes	Using existing critical review forms (qualitative and quantitative research)
10. Did the review authors report on the sources of funding for the studies included in the review?	No	
11. If meta-analyses was performed did the review authors use appropriate methods for statistical combination of results?	NA	
12. If meta-analysis was performed, did the review authors assess the potential impact of risk of bias in individual studies on the results of the meta-analysis or other evidence synthesis?	NA	

13. Did the review authors account for risk of bias in individual studies when interpreting/discussing the results of the review?	No	
14. Did the review authors provide a satisfactory explanation for, and discussion of, any heterogeneity observed in the results of the review?	No	
15. If they performed quantitative synthesis did the review authors carry out an adequate investigation of publication bias (small study bias) and discuss its likely impact on the results of the review?	NA	
16. Did the review authors report any potential sources of conflict of interest, including any funding they received for conducting the review?	Yes	

**Author, publication year: Douma, 2017**		
**Item**	**Yes, partial yes or no**	**Explanation**
1. Did the research questions and inclusion criteria for the review include the components of PICO?	Yes	
2. Did the report of the review contain an explicit statement that the review methods were established prior to the conduct of the review and did the report justify any significant deviations from the protocol?	No	
3. Did the review authors explain their selection of the study designs for inclusion in the review?	Yes	
4. Did the review authors use a comprehensive literature search strategy?	Partial yes	No grey literature
5. Did the review authors perform study selection in duplicate?	Yes	
6. Did the review authors perform data extraction in duplicate?	No	
7. Did the review authors provide a list of excluded studies and justify the exclusions?	No	
8. Did the review authors describe the included studies in adequate detail?	Yes	
9. Did the review authors use a satisfactory technique for assessing the risk of bias in individual studies that were included in the review?	Yes	MMAT tool
10. Did the review authors report on the sources of funding for the studies included in the review?	No	
11. If meta-analyses was performed did the review authors use appropriate methods for statistical combination of results?	NA	
12. If meta-analysis was performed, did the review authors assess the potential impact of risk of bias in individual studies on the results of the meta-analysis or other evidence synthesis?	NA	
13. Did the review authors account for risk of bias in individual studies when interpreting/discussing the results of the review?	Partial yes	
14. Did the review authors provide a satisfactory explanation for, and discussion of, any heterogeneity observed in the results of the review?	No	
15. If they performed quantitative synthesis did the review authors carry out an adequate investigation of publication bias (small study bias) and discuss its likely impact on the results of the review?	NA	
16. Did the review authors report any potential sources of conflict of interest, including any funding they received for conducting the review?	Yes	

**Author, publication year: Jacinto, 2021**		
**Item**	**Yes, partial yes or no**	**Explanation**
1. Did the research questions and inclusion criteria for the review include the components of PICO?	Yes	
2. Did the report of the review contain an explicit statement that the review methods were established prior to the conduct of the review and did the report justify any significant deviations from the protocol?	Yes	INPLASY2021100092
3. Did the review authors explain their selection of the study designs for inclusion in the review?	No	Criteria: review articles, comments, theses or

		abstracts published in minutes of congresses or conferences
4. Did the review authors use a comprehensive literature search strategy?	Partial yes	
5. Did the review authors perform study selection in duplicate?	Yes	
6. Did the review authors perform data extraction in duplicate?	No	
7. Did the review authors provide a list of excluded studies and justify the exclusions?	No	
8. Did the review authors describe the included studies in adequate detail?	Yes	
9. Did the review authors use a satisfactory technique for assessing the risk of bias in individual studies that were included in the review?	Yes	Downs and Black Scale
10. Did the review authors report on the sources of funding for the studies included in the review?	No	
11. If meta-analyses was performed did the review authors use appropriate methods for statistical combination of results?	NA	
12. If meta-analysis was performed, did the review authors assess the potential impact of risk of bias in individual studies on the results of the meta-analysis or other evidence synthesis?	NA	
13. Did the review authors account for risk of bias in individual studies when interpreting/discussing the results of the review?	No	
14. Did the review authors provide a satisfactory explanation for, and discussion of, any heterogeneity observed in the results of the review?	No	
15. If they performed quantitative synthesis did the review authors carry out an adequate investigation of publication bias (small study bias) and discuss its likely impact on the results of the review?	NA	
16. Did the review authors report any potential sources of conflict of interest, including any funding they received for conducting the review?	Yes	

**Author, publication year: Laxton, 2023**		
**Item**	**Yes, partial yes or no**	**Explanation**
1. Did the research questions and inclusion criteria for the review include the components of PICO?	Yes	
2. Did the report of the review contain an explicit statement that the review methods were established prior to the conduct of the review and did the report justify any significant deviations from the protocol?	No	
3. Did the review authors explain their selection of the study designs for inclusion in the review?	No	
4. Did the review authors use a comprehensive literature search strategy?	Partial yes	References lists manually checked. No grey literature
5. Did the review authors perform study selection in duplicate?	No	
6. Did the review authors perform data extraction in duplicate?	Yes	Agreement 95%
7. Did the review authors provide a list of excluded studies and justify the exclusions?	No	
8. Did the review authors describe the included studies in adequate detail?	Yes	
9. Did the review authors use a satisfactory technique for assessing the risk of bias in individual studies that were included in the review?	Yes	CASP checklist + NIH's Quality Assessment Tool
10. Did the review authors report on the sources of funding for the studies included in the review?		
11. If meta-analyses was performed did the review authors use appropriate methods for statistical combination of results?	NA	
12. If meta-analysis was performed, did the review authors assess the potential impact of risk of bias in individual studies on the results of the meta-analysis or other evidence synthesis?	NA	
13. Did the review authors account for risk of bias in individual studies when interpreting/discussing the results of the review?	Partial yes	
14. Did the review authors provide a satisfactory explanation for, and discussion of, any heterogeneity observed in the results of the review?	No	

15. If they performed quantitative synthesis did the review authors carry out an adequate investigation of publication bias (small study bias) and discuss its likely impact on the results of the review?	NA	
16. Did the review authors report any potential sources of conflict of interest, including any funding they received for conducting the review?	No	

Author, publication year: MacDonald, 2021		
**Item**	**Yes, partial yes or no**	**Explanation**
1. Did the research questions and inclusion criteria for the review include the components of PICO?	Yes	
2. Did the report of the review contain an explicit statement that the review methods were established prior to the conduct of the review and did the report justify any significant deviations from the protocol?	No	
3. Did the review authors explain their selection of the study designs for inclusion in the review?	No	
4. Did the review authors use a comprehensive literature search strategy?	No	Search strategy on request, no grey literature
5. Did the review authors perform study selection in duplicate?	Yes	
6. Did the review authors perform data extraction in duplicate?	No	Agreement unknown
7. Did the review authors provide a list of excluded studies and justify the exclusions?	No	
8. Did the review authors describe the included studies in adequate detail?	No	
9. Did the review authors use a satisfactory technique for assessing the risk of bias in individual studies that were included in the review?	Yes	MMAT tool
10. Did the review authors report on the sources of funding for the studies included in the review?	No	
11. If meta-analyses was performed did the review authors use appropriate methods for statistical combination of results?	NA	
12. If meta-analysis was performed, did the review authors assess the potential impact of risk of bias in individual studies on the results of the meta-analysis or other evidence synthesis?	NA	
13. Did the review authors account for risk of bias in individual studies when interpreting/discussing the results of the review?	No	
14. Did the review authors provide a satisfactory explanation for, and discussion of, any heterogeneity observed in the results of the review?	No	
15. If they performed quantitative synthesis did the review authors carry out an adequate investigation of publication bias (small study bias) and discuss its likely impact on the results of the review?	NA	
16. Did the review authors report any potential sources of conflict of interest, including any funding they received for conducting the review?	Yes	

Author, publication year: Maurer, 2019		
**Item**	**Yes, partial yes or no**	**Explanation**
1. Did the research questions and inclusion criteria for the review include the components of PICO?	Yes	
2. Did the report of the review contain an explicit statement that the review methods were established prior to the conduct of the review and did the report justify any significant deviations from the protocol?	No	
3. Did the review authors explain their selection of the study designs for inclusion in the review?	No	
4. Did the review authors use a comprehensive literature search strategy?	Partial yes	References lists checked
5. Did the review authors perform study selection in duplicate?	No	
6. Did the review authors perform data extraction in duplicate?	No	

7. Did the review authors provide a list of excluded studies and justify the exclusions?	No	
8. Did the review authors describe the included studies in adequate detail?	Partial yes	
9. Did the review authors use a satisfactory technique for assessing the risk of bias in individual studies that were included in the review?	Yes	CASP tool
10. Did the review authors report on the sources of funding for the studies included in the review?	No	
11. If meta-analyses was performed did the review authors use appropriate methods for statistical combination of results?	NA	
12. If meta-analysis was performed, did the review authors assess the potential impact of risk of bias in individual studies on the results of the meta-analysis or other evidence synthesis?	NA	
13. Did the review authors account for risk of bias in individual studies when interpreting/discussing the results of the review?	No	
14. Did the review authors provide a satisfactory explanation for, and discussion of, any heterogeneity observed in the results of the review?	No	
15. If they performed quantitative synthesis did the review authors carry out an adequate investigation of publication bias (small study bias) and discuss its likely impact on the results of the review?	NA	
16. Did the review authors report any potential sources of conflict of interest, including any funding they received for conducting the review?	Yes	

**Author, publication year: Narsakka (2022)**		
**Item**	**Yes, partial yes or no**	**Explanation**
1. Did the research questions and inclusion criteria for the review include the components of PICO?	Yes	
2. Did the report of the review contain an explicit statement that the review methods were established prior to the conduct of the review and did the report justify any significant deviations from the protocol?	Yes	
3. Did the review authors explain their selection of the study designs for inclusion in the review?	No	
4. Did the review authors use a comprehensive literature search strategy?	Partial yes	References lists were screened
5. Did the review authors perform study selection in duplicate?	Yes	
6. Did the review authors perform data extraction in duplicate?	No	
7. Did the review authors provide a list of excluded studies and justify the exclusions?	No	
8. Did the review authors describe the included studies in adequate detail?	Yes	
9. Did the review authors use a satisfactory technique for assessing the risk of bias in individual studies that were included in the review?	Yes	Joanna Briggs Institute's Critical Appraisal Checklists
10. Did the review authors report on the sources of funding for the studies included in the review?	No	
11. If meta-analyses was performed did the review authors use appropriate methods for statistical combination of results?	NA	
12. If meta-analysis was performed, did the review authors assess the potential impact of risk of bias in individual studies on the results of the meta-analysis or other evidence synthesis?	NA	
13. Did the review authors account for risk of bias in individual studies when interpreting/discussing the results of the review?	No	
14. Did the review authors provide a satisfactory explanation for, and discussion of, any heterogeneity observed in the results of the review?	No	
15. If they performed quantitative synthesis did the review authors carry out an adequate investigation of publication bias (small study bias) and discuss its likely impact on the results of the review?	NA	
16. Did the review authors report any potential sources of conflict of interest, including any funding they received for conducting the review?	Yes	

Author, publication year: Vseteckova, 2018		
**Item**	**Yes, partial yes or no**	**Explanation**
1. Did the research questions and inclusion criteria for the review include the components of PICO?	Yes	
2. Did the report of the review contain an explicit statement that the review methods were established prior to the conduct of the review and did the report justify any significant deviations from the protocol?	No	
3. Did the review authors explain their selection of the study designs for inclusion in the review?	Yes	
4. Did the review authors use a comprehensive literature search strategy?	Partial yes	Lots of databases. Reference lists checked.
5. Did the review authors perform study selection in duplicate?	No	
6. Did the review authors perform data extraction in duplicate?	No	Critical Appraisal Skills Programme (CASP) tool used
7. Did the review authors provide a list of excluded studies and justify the exclusions?	No	
8. Did the review authors describe the included studies in adequate detail?	Partial yes	
9. Did the review authors use a satisfactory technique for assessing the risk of bias in individual studies that were included in the review?	No	Only for RCTs: 'Risk of Bias' tool
10. Did the review authors report on the sources of funding for the studies included in the review?	No	
11. If meta-analyses was performed did the review authors use appropriate methods for statistical combination of results?	NA	
12. If meta-analysis was performed, did the review authors assess the potential impact of risk of bias in individual studies on the results of the meta-analysis or other evidence synthesis?	NA	
13. Did the review authors account for risk of bias in individual studies when interpreting/discussing the results of the review?	No	
14. Did the review authors provide a satisfactory explanation for, and discussion of, any heterogeneity observed in the results of the review?	No	
15. If they performed quantitative synthesis did the review authors carry out an adequate investigation of publication bias (small study bias) and discuss its likely impact on the results of the review?	NA	
16. Did the review authors report any potential sources of conflict of interest, including any funding they received for conducting the review?	Yes	

Reference list of included studies in the systematic reviews

Physical activities

1. Brooker, K.; Van Dooren, K.; McPherson, L.; Lennox, N.; Ware, R. Systematic Review of Interventions Aiming to Improve Involvement in Physical Activity Among Adults With Intellectual Disability. *Journal of Physical Activity & Health* **2015**, 12(3), 434–444, https://doi.org/10.1123/jpah.2013-0014
2. Barrett, E.; Casey, B.; Dollard, M.; McCarthy, B.; Casey, D. Effectiveness of Functionally Based Physical Activity Programs on Physical, Psychological, Cognitive, and Adverse Outcomes in Older Adults Living in Nursing Homes: Systematic Review. *Activities, Adaptation & Aging* **2020**, 45(4), 306–347, https://doi.org/10.1080/01924788.2020.1794352
3. Brett, L.; Traynor, V.; Stapley, P. Effects of Physical Exercise on Health and Well-Being of Individuals Living With a Dementia in Nursing Homes: A Systematic Review. *Journal of the American Medical Directors Association* **2016**, 17(2), 104–116, https://doi.org/10.1016/j.jamda.2015.08.016
4. Jansen, C.; Claßen, K.; Wahl, H.; Hauer, K. Effects of Interventions on Physical Activity in Nursing Home Residents. *European Journal of Ageing* **2015**, 12(3), 261–271, https://doi.org/10.1007/s10433-015-0344-1
5. Temple, V.A.; Frey, G.C.; Stanish, H.I. Interventions to Promote Physical Activity for Adults With Intellectual Disabilities. *Salud Pública de México* **2017**, 59(4), 446, https://doi.org/10.21149/8218
6. Wylie, G.; Kroll, T.; Witham, M.D.; Morris, J. Increasing Physical Activity Levels in Care Homes for Older People: A Quantitative Scoping Review of Intervention Studies to Guide Future Research. *Disability and Rehabilitation* **2022**, 45(19), 3160–3176, https://doi.org/10.1080/09638288.2022.2118869

Technology

1. Agbangla, N.F.; Séba, M.P.; Bunlon, F. Snacktivity™, Giant Games and Immersive Virtual Reality Exercises: A Rapid Narrative Review of These New Physical Activity Practices Among Older People Living in Nursing Homes and Long-Term Care Facilities. *Healthcare* **2022**, 10(10). https://pubmed.ncbi.nlm.nih.gov/36292344/
2. Chen, P.J.; Hsu, H.F.; Chen, K.M.; Belcastro, F. VR Exergame Interventions Among Older Adults Living in Long-Term Care Facilities: A Systematic Review With Meta-Analysis. *Annals of Physical and Rehabilitation Medicine* **2023**, 66(3), 101702, https://pubmed.ncbi.nlm.nih.gov/36028201/
3. Chu, C.H.; Quan, A.M.L.; Souter, A.; Krisnagopal, A.; Biss, R.K. Effects of Exergaming on Physical and Cognitive Outcomes of Older Adults Living in Long-Term Care Homes: A Systematic Review. *Gerontology* **2022**, 68(9), https://pubmed.ncbi.nlm.nih.gov/35290983/
4. Diener, J.; Rayling, S.; Bezold, J.; Krell-Roesch, J.; Woll, A.; Wunsch, K. Effectiveness and Acceptability of e- and m-Health Interventions to Promote Physical Activity and Prevent Falls in Nursing Homes—A Systematic Review. *Frontiers in Physiology* **2022**, 13, https://doi.org/10.3389/fphys.2022.894397
5. Kukkohovi, S.; Siira, H.; Arolaakso, S.; Miettunen, J.; Elo, S. The Effectiveness of Digital Gaming on the Functioning and Activity of Older People Living in Long-Term Care Facilities: A Systematic Review and Meta-Analysis. *Aging Clinical and Experimental Research* **2023**, 35(8), 1595–1608, https://doi.org/10.1007/s40520-023-02459-y
6. Lancioni, G.E.; Singh, N.N.; O'Reilly, M.; Sigafoos, J.; Alberti, G.; Desideri, L. Programs Using Stimulation-Regulating Technologies to Promote Physical Activity in People With Intellectual and Multiple Disabilities: Scoping Review. *JMIR Rehabilitation and Assistive Technologies* **2022**, 9(2), e35217, https://pubmed.ncbi.nlm.nih.gov/35389365/
7. Li, X.; Huang, J.; Kong, Z.; Sun, F.; Sit, C.H.P.; Li, C. Effects of Virtual Reality-Based Exercise on Physical Fitness in People With Intellectual Disability: A Systematic Review of Randomized Controlled Trials. *Games for Health Journal* **2023**, 12(2), 89–99, https://doi.org/10.1089/g4h.2022.0168
8. Swinnen, N.; Vandenbulcke, M.; Vancampfort, D. Exergames in People With Major Neurocognitive Disorder: A Systematic Review. *Disability and Rehabilitation: Assistive Technology* **2022**, 17(4), 376–389, https://pubmed.ncbi.nlm.nih.gov/32697614/

Motivation

1. Aitchison, B.; Rushton, A.B.; Martin, P.; Barr, M.; Soundy, A.; Heneghan, N.R. The Experiences and Perceived Health Benefits of Individuals With a Disability Participating in Sport: A Systematic Review and Narrative Synthesis. *Disability and Health Journal* **2022**, 15(1), 101164.
2. Anderiesen, H.; Scherder, E.J.A.; Goossens, R.H.M.; Sonneveld, M.H. A Systematic Review—Physical Activity in Dementia: The Influence of the Nursing Home Environment. *Applied Ergonomics* **2014**, 45(6), 1678–1686.
3. Benjamin, K.; Edwards, N.; Ploeg, J.; Legault, F. Barriers to Physical Activity and Restorative Care for Residents in Long-Term Care: A Review of the Literature. *Journal of Aging and Physical Activity* **2014**, 22(1), 154–165.
4. Bossink, L.W.M.; Van der Putten, A.A.; Vlaskamp, C. Understanding Low Levels of Physical Activity in People With Intellectual Disabilities: A Systematic Review to Identify Barriers and Facilitators. *Research in Developmental Disabilities* **2017**, 68, 95–110.
5. Douma, J.G.; Volkers, K.M.; Engels, G.; Sonneveld, M.H.; Goossens, R.H.M.; Scherder, E.J.A. Setting-Related Influences on Physical Inactivity of Older Adults in Residential Care Settings: A Review. *BMC Geriatrics* **2017**, 17(1), 1–10, https://doi.org/10.1186/s12877-017-0487-3
6. Jacinto, M.; Vitorino, A.S.; Palmeira, D.; Antunes, R.; Ferreira, J.P.; Bento, T.; et al. Perceived Barriers of Physical Activity Participation in Individuals With Intellectual Disability—A Systematic Review. *Healthcare* **2021**, 9(11).
7. Laxton, P.; Patterson, F.; Healy, S. Factors Related to Physical Activity in Adults With Intellectual Disabilities in Group Home Settings: A Systematic Literature Review. *Adapted Physical Activity Quarterly* **2023**, 40(2), 347–377.
8. MacDonald, C.; Bush, P.L.; Foley, J.T. Physical Activity Promotion and Adults With Intellectual Disabilities: A Neglected Area. *Journal of Intellectual Disabilities* **2021**, 26(2), 509–521, https://doi.org/10.1177/1744629521995345
9. Maurer, C.; Draganescu, S.; Mayer, H.; Gattinger, H. Attitudes and Needs of Residents in Long-Term Care Facilities Regarding Physical Activity—A Systematic Review and Synthesis of Qualitative Studies. *Journal of Clinical Nursing* **2019**, 28(13–14), 2386–2400, https://pubmed.ncbi.nlm.nih.gov/30589972/
10. Narsakka, N.; Suhonen, R.; Kielo-Viljamaa, E.; Stolt, M. Physical, Social, and Symbolic Environment Related to Physical Activity of Older Individuals in Long-Term Care: A Mixed-Method Systematic Review. *International Journal of Nursing Studies* **2022**, 135, 104350.
11. Vseteckova, J.; Dadova, K.; Gracia, R.; Ryan, G.; Borgstrom, E.; Abington, J.; et al. Barriers and Facilitators to Adherence to Walking Group Exercise in Older People Living With Dementia in the Community: A Systematic Review. *European Review of Aging and Physical Activity* **2020**, 17(1).
